# Pervasive downstream RNA hairpins dynamically dictate start-codon selection

**DOI:** 10.1038/s41586-023-06500-y

**Published:** 2023-09-06

**Authors:** Yezi Xiang, Wenze Huang, Lianmei Tan, Tianyuan Chen, Yang He, Patrick S. Irving, Kevin M. Weeks, Qiangfeng Cliff Zhang, Xinnian Dong

**Affiliations:** 1 Department of Biology, Duke University, Durham, NC USA; 2 Howard Hughes Medical Institute, Duke University, Durham, NC USA; 3 MOE Key Laboratory of Bioinformatics, Center for Synthetic and Systems Biology, School of Life Sciences, Tsinghua University, Beijing, China; 4 Beijing Frontier Research Center for Biological Structures, Beijing Advanced Innovation Center for Structural Biology, Tsinghua University, Beijing, China; 5 Tsinghua-Peking Center for Life Sciences, Beijing, China; 6 Department of Pharmacology and Cancer Biology, Duke Medical Center, Duke University, Durham, NC USA; 7 Department of Chemistry, University of North Carolina, Chapel Hill, NC USA

**Keywords:** Molecular biology, Plant sciences

## Abstract

Translational reprogramming allows organisms to adapt to changing conditions. Upstream start codons (uAUGs), which are prevalently present in mRNAs, have crucial roles in regulating translation by providing alternative translation start sites^1–4^. However, what determines this selective initiation of translation between conditions remains unclear. Here, by integrating transcriptome-wide translational and structural analyses during pattern-triggered immunity in *Arabidopsis*, we found that transcripts with immune-induced translation are enriched with upstream open reading frames (uORFs). Without infection, these uORFs are selectively translated owing to hairpins immediately downstream of uAUGs, presumably by slowing and engaging the scanning preinitiation complex. Modelling using deep learning provides unbiased support for these recognizable double-stranded RNA structures downstream of uAUGs (which we term uAUG-ds) being responsible for the selective translation of uAUGs, and allows the prediction and rational design of translating uAUG-ds. We found that uAUG-ds-mediated regulation can be generalized to human cells. Moreover, uAUG-ds-mediated start-codon selection is dynamically regulated. After immune challenge in plants, induced RNA helicases that are homologous to Ded1p in yeast and DDX3X in humans resolve these structures, allowing ribosomes to bypass uAUGs to translate downstream defence proteins. This study shows that mRNA structures dynamically regulate start-codon selection. The prevalence of this RNA structural feature and the conservation of RNA helicases across kingdoms suggest that mRNA structural remodelling is a general feature of translational reprogramming.

## Abstract (web summary)

Double-stranded RNA structures downstream of start codons play a role in translation initiation by regulating start-codon selection in plant immune responses, and also contribute to translational reprogramming in mammalian systems.

## Main

Translation of eukaryotic genes is regulated by multiple features in mRNAs. Among them, uAUGs and associated uORFs are widely present in the 5′ leader sequences (around 64% in humans and around 54% in *Arabidopsis*)^3^. Most eukaryotic mRNAs are translated in a cap-dependent manner, with the 43S preinitiation complex scanning from the 5′ cap and initiating translation at a start codon by recruiting the 60S ribosomal subunit^5–7^. The presence of uAUGs provides potential alternative sites for the preinitiation complex to start translation before it reaches the main AUG (mAUG); and if translation initiates from uAUGs, it typically inhibits translation from downstream mAUGs^2,8–10^. This inhibitory role of uAUGs is crucial for controlling the production of specific proteins in normal conditions, particularly those involved in the stress response or in cell death^11–13^. For example, constitutive translation of the key plant immune transcription factor TL1-binding factor (TBF1; *AT4G36990.1*) without the two uAUGs and uORFs in its 5′ leader sequence causes lethality^14^. Notably, most uORFs do not have conserved primary sequences despite undergoing positive Darwinian selection^3^, which suggests that they inhibit the translation of main ORFs (mORFs) mostly through competition for ribosomes rather than through their translational products^1^.

uORF-mediated inhibition can be alleviated in a variety of conditions^4,8,15,16^, permitting the translation of downstream mORFs. This translational switch from uORF to mORF has been well studied in a few transcription factors, including yeast Gcn4 and mammalian ATF4, through stress-induced phosphorylation and inactivation of eukaryotic translation initiation factor 2α (eIF2α)^11,13^. However, inactivation of eIF2α leads to a global shutdown of translation, which, although essential for some stress responses (for example, nutrient deprivation^11^), is deleterious and absent during most eukaryotic developmental stages or in abiotic and biotic stress conditions^17–20^ (for example, immune responses in plants, such as pattern-triggered immunity; PTI^20^). This raises the fundamental question of what mRNA features, in conjunction with the translational machinery, dynamically dictate from which AUG to initiate translation and consequently control protein production under different conditions.

## Translational switch after immune induction

**Fig. 1:**
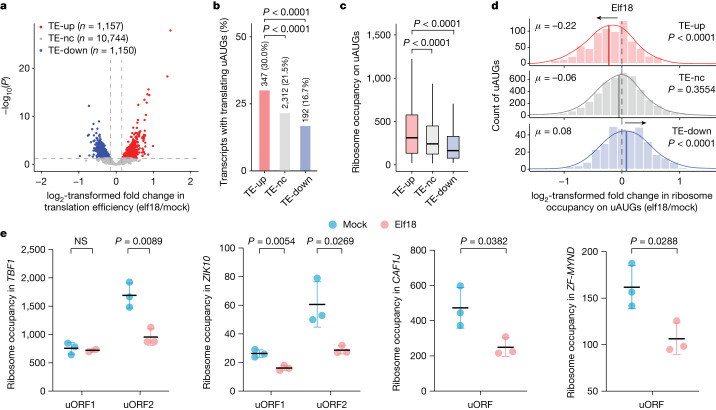
Translational dynamics of uORF-containing transcripts. **a**, Volcano plot of global changes in translational efficiency during PTI. TE-up, transcripts with upregulated translational efficiency (*P* < 0.05, log_2_-transformed fold change > 0.16); TE-nc, transcripts with no changes in translational efficiency (*P* > 0.05); TE-down, transcripts with downregulated translational efficiency (*P* < 0.05, log_2_-transformed fold change < –0.16). **b**, Number and percentage of transcripts with translating uAUGs in the TE-up, TE-nc and TE-down groups. Two-tailed Fisher’s exact test was used to determine the *P* value of the difference between groups. **c**, Box plot of ribosome occupancy (normalized read counts) on translating uAUGs in the TE-up (*n* = 347), TE-nc (*n* = 2,312), and TE-down (*n* = 192) transcripts in the mock condition. *P* values were calculated by two-tailed Mann–Whitney tests. Boxes, interquartile range (IQR); centre lines, median; whiskers, values within 1.5 × IQR of the top and bottom quartiles. **d**, Histograms with density curves of log_2_-transformed fold change of ribosome occupancy on translating uAUGs in the TE-up, TE-nc and TE-down transcripts in response to elf18 treatment. *μ*, average log_2_ transformed fold change value. *P* values were calculated by two-tailed paired *t*-tests. **e**, Ribosome occupancy on the uORF(s) in four TE-up transcripts, namely *TBF1*, *ZIK10*, *CAF1J* and *ZF-MYND* (*AT1G70160.1*), in response to mock and elf18 treatment. *P* values were calculated by two-tailed Student’s *t*-tests. NS, not significant. Values are mean ± s.d. (*n* = 3 independent biological replicates).

**Extended Data Fig. 1:**
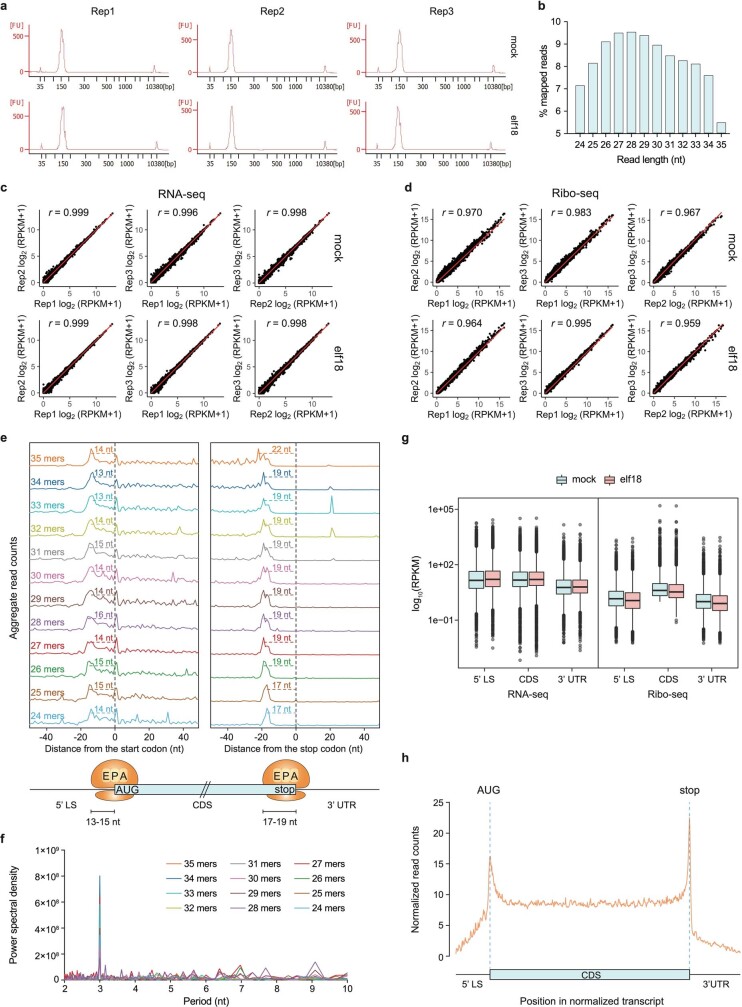
Quality and reproducibility of RNA-seq and Ribo-seq data. **a**, BioAnalyzer profiles showed high quality of the Ribo-seq libraries. Apart from the internal standard sized at 35 bp and 10,380 bp, a single peak at around 150 bp was present in all the libraries for mock and elf18 treatment in all three biological replicates (Reps 1–3). **b**, Length distribution of reads from the Ribo-seq libraries. **c**,**d**, Correlations among the three replicates of RNA-seq (**c**) and Ribo-seq (**d**) data from mock- and elf18-treated samples. Data are shown as correlations of log_2_(RPKM+1) for all the genes. *r*, Pearson correlation coefficient. **e**, Metagene analysis on the average read counts surrounding start and stop codons for reads at different lengths (top). P-site offsets were detected at the length of 13–15 nt surrounding start codons and at the length of 17–19 nt surrounding stop codons (bottom). 5′ LS, 5′ leader sequence. **f**, Power spectral density of normalized Ribo-seq read counts in the 300-nt window downstream of the start codon shows 3-nt periodicity. The units are (normalized read counts)^2 per nucleotide period. **g**, Total RNA-seq and Ribo-seq read distribution in 5′ LS, CDS, and 3′ UTR of the 13,051 expressed transcripts (*n* = 13,051). Boxes, IQR. Centre lines, median. Whiskers, values within 1.5 × IQR of the top and bottom quartiles. Grey circles represent RPKM values for individual outlier transcripts. **h**, Metagene analysis across normalized transcript for Ribo-seq reads in all the mock and elf18-induced samples with the read length ranging from 24 nt to 35 nt. 5′ LS, 5′ leader sequence. 3′ UTR, 3′ untranslated region.

**Extended Data Fig. 2:**
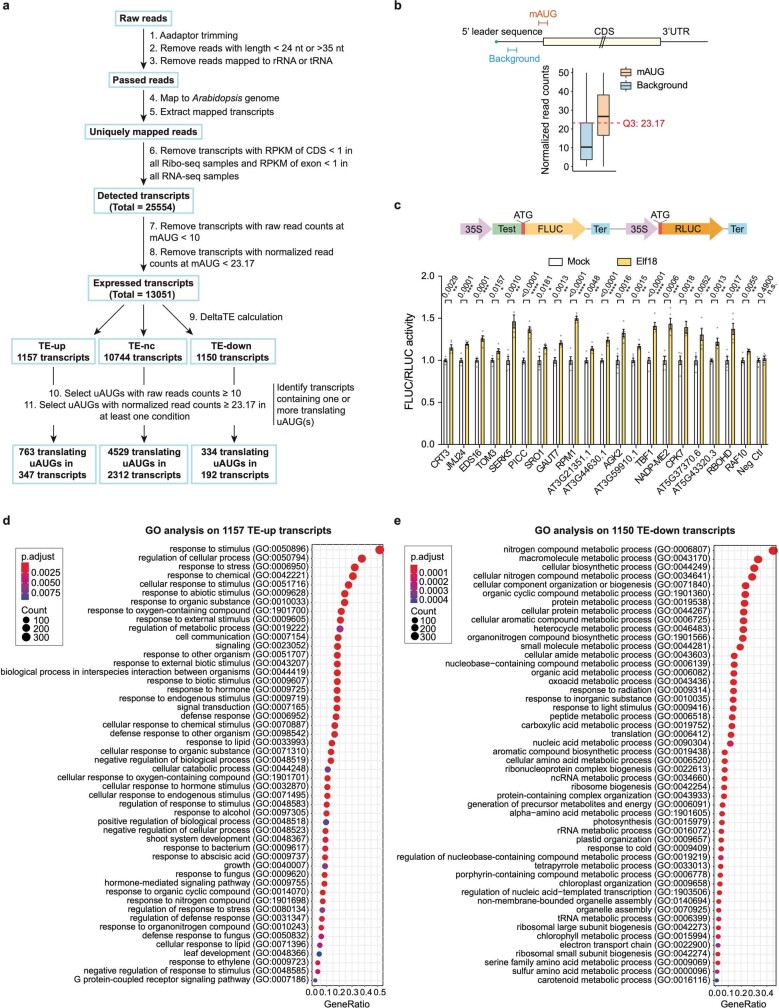
Global analysis of translational dynamics and dual-luciferase reporter study of uAUG-containing transcripts. **a**, Flow chart of RNA-seq and Ribo-seq data analysis. **b**, Strategy for the identification of translating mAUGs and uAUGs (see Methods for details). **c**, Dual-luciferase reporter study (top) of translational responses of the 5′ leader sequences of 20 TE-up transcripts to elf18 induction (bottom). FLUC reporter without the inserted test sequence was used as a negative control (Neg Ctl). *P* values were calculated by two-tailed Student’s *t*-test. Values are mean ± s.e.m. (*n* = 5 biological replicates). **d**,**e**, GO enrichment analysis on the 1,157 TE-up transcripts (**d**) and 1,150 TE-down transcripts (**e**). The size of the dot represents the number of genes that fall into each group. The colour of the dot represents adjusted *P* value.

To identify mechanisms involved in the uAUG-mediated regulation of translation, we first performed global ribosome sequencing (Ribo-seq; sequencing of ribosome-protected RNA fragments) in *Arabidopsis* seedlings in response to the induction of PTI by elf18 (N-terminal epitope of the bacterial elongation factor Tu)^21^. The optimized Ribo-seq pipeline had a sufficiently high resolution to examine the translational activities in 5′ leader sequences (Methods and Extended Data Fig. 1). Comparing elf18-treated samples to mock-treated controls, we identified, among the 13,051 expressed transcripts, 1,157 with increased translational efficiency (TE-up), 1,150 with decreased translational efficiency (TE-down), and the rest with no significant changes in translational efficiency (TE-nc) (Fig. 1a and Extended Data Fig. 2a,b). We selected 20 TE-up transcripts and used their 5′ leader sequences to drive the translation of the constitutively transcribed firefly luciferase (FLUC) reporter. Using the constitutively expressed Renilla luciferase (RLUC) as a control, this ‘dual luciferase’ assay^22^ confirmed the elf18-induced translation (Extended Data Fig. 2c) observed in the Ribo-seq results. Gene Ontology (GO) analysis^23–25^ of the TE-up genes revealed an enrichment of biological processes in response to a variety of environmental stresses, such as biotic stimuli, abiotic stimuli and chemicals, whereas GO terms for the TE-down genes were mostly growth-related metabolic processes (Extended Data Fig. 2d,e). Because the TE-up category includes key immune transcription factors, such as TBF1, a moderate increase in their translation could have a substantial effect on the downstream defence response.

To systematically identify uAUGs that can be recognized by the preinitiation complex and initiate translation (‘translating uAUGs’), we focused on those uAUGs with ribosomal associations above the background levels (Methods and Extended Data Fig. 2a,b). We identified 5,626 translating uAUGs across the 13,051 expressed transcripts, with some transcripts having multiple translating uAUGs. Notably, we discovered that translating uAUGs were significantly enriched in the TE-up transcripts (30.0%), compared to the TE-nc (21.5%) and TE-down mRNAs (16.7%) (Fig. 1b). This finding suggests that translation initiation from uAUGs has a general role in regulating immune-associated translation.

Next, we examined the global translational dynamics of the translating uAUGs. In the mock condition, translating uAUGs in the TE-up transcripts had significantly higher ribosomal associations than did those in the TE-nc and TE-down transcripts (Fig. 1c), suggesting higher rates of translation initiation from these uAUGs in the TE-up transcripts without immune induction (mock). After treatment with elf18, there was a significant decrease in ribosomal association with these translating uAUGs in the TE-up transcripts, whereas this reduction was not observed in the TE-nc and TE-down transcripts (Fig. 1d). Closer examination of the Ribo-seq data for a few TE-up transcripts, including *TBF1* (refs ^14,19^), showed that there was a significant reduction in ribosome occupancy on the inhibitory uORFs (uORF2 for *TBF1* and *ZIK10*) in response to elf18 treatment (Fig. 1e). Because translation initiation from uAUGs typically inhibits the downstream mORF translation^2,8–10^, this elf18-triggered reduction in uAUG translation suggests an immune-induced release of the uAUG-mediated inhibition of downstream mORF translation. Collectively, our global characterization (Fig. 1c,d) and direct analysis on marker genes (Fig. 1e) revealed the common regulatory dynamics of translating uAUGs in the TE-up transcripts: they are preferentially recognized and translated under the mock condition, but are bypassed to permit translation initiation from mAUGs in response to immune induction.

## Downstream hairpins dictate AUG selection

**Fig. 2:**
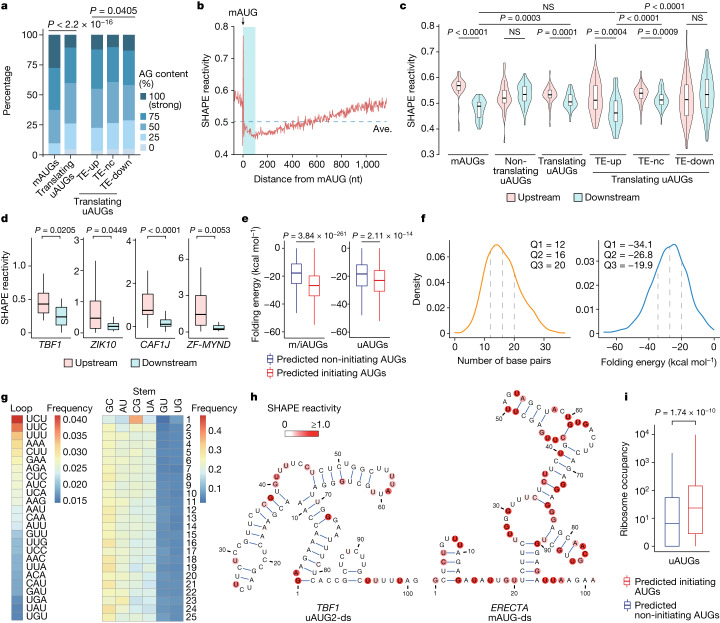
Global SHAPE-MaP and deep learning analyses reveal hairpin structures downstream of mAUGs and uAUGs that have a role in dictating translation initiation. **a**, Kozak sequence contexts (AG content) flanking mAUGs and translating uAUGs. *P* values were calculated by two-sided chi-squared test. **b**, Average SHAPE reactivities across all expressed transcripts aligned by start codons of CDS in the mock condition. Red line, average reactivity for every three nucleotides. Ave., average SHAPE reactivity across all of the nucleotides. Blue shading, 100 nt downstream of mAUG. **c**, Violin plots showing comparisons of SHAPE reactivities 50 nt upstream and 50 nt downstream of mAUGs or uAUGs in the mock condition. **d**, Box plots showing differences in SHAPE reactivities 50 nt upstream and 50 nt downstream of translating uAUGs in four TE-up transcripts. Only the major inhibitory uAUGs (that is, uAUG2s in *TBF1* and *ZIK10*) are shown. **e**, Box plots showing the differences in folding energy of RNA secondary structures downstream of predicted initiating and non-initiating AUGs. m/iAUGs, mAUGs and internal AUGs. **f**, Distributions of base-pair numbers and folding energies of RNA secondary structures downstream of predicted initiating AUGs. **g**, Heat maps showing the frequencies of nucleotides in the loop and the stem of hairpin structures downstream of predicted initiating AUGs that are significantly distinct from the background (*P* = 1.4 × 10^–109^ for the loop and *P* = 1.5 × 10^–79^ for the stem, calculated by chi-squared test). Numbers 1 to 25 show the position of each base pair, which were counted starting from the end of the loop. **h**, Models of RNA secondary structures downstream of uAUG2 (uAUG2-ds) of *TBF1* and mAUG (mAUG-ds) of *ERECTA*. **i**, Box plot showing the difference in ribosome occupancy on predicted initiating and non-initiating uAUGs. For **c**–**e**,**i**, boxes, IQR; centre lines, median; whiskers, values within 1.5 × IQR of the top and bottom quartiles. *P* values were calculated by two-tailed Mann–Whitney tests.

To address the question of how start codons are dynamically selected to initiate translation in different conditions, we first assessed the Kozak sequence context flanking the AUGs (–3 to +4, with A in AUG being +1) which is known to affect the recognition of start codons by the translation preinitiation complex^26^. A previous analysis suggested that in plants, a higher adenine and guanine (AG) content is associated with higher translational activity^27^. Using this criterion, we assessed the Kozak contexts for the uAUGs and mAUGs in all the expressed transcripts. We found that mAUGs have markedly higher AG contents than do translating uAUGs (Fig. 2a), in agreement with previous studies in animals, which found that mAUGs generally have more preferable Kozak sequence contexts than do uAUGs^28,29^. However, the Kozak contexts for translating uAUGs among the TE-up, TE-nc and TE-down transcripts are similar (Fig. 2a), suggesting that although the Kozak sequence context is important for start-codon recognition in static conditions, it is unlikely to be responsible for the elf18-mediated switch from uAUG to mAUG translation in the TE-up transcripts.

**Extended Data Fig. 3:**
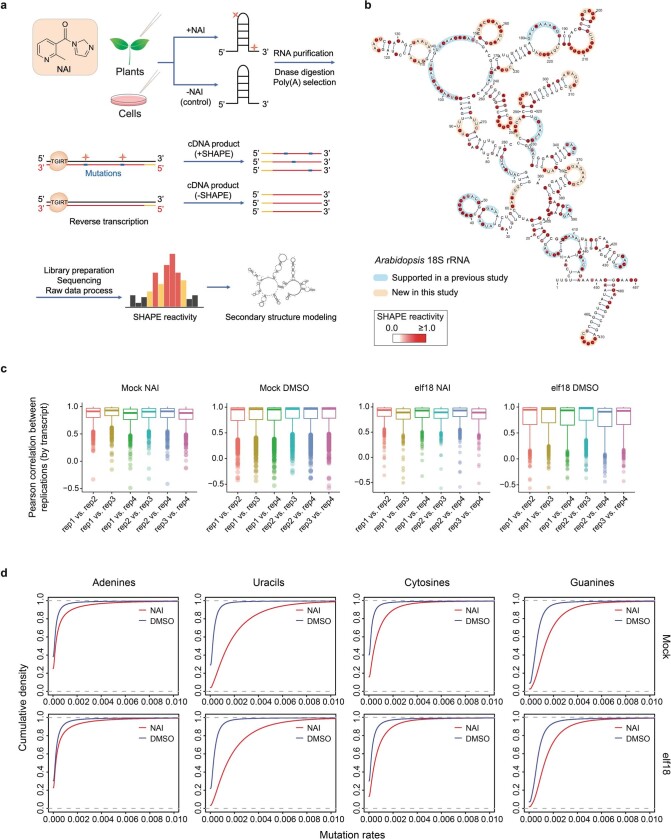
Quality and reproducibility of global and targeted in planta SHAPE-MaP. **a**, Flow chart of in planta SHAPE-MaP protocol. **b**, Comparison of in vivo *Arabidopsis* 18S rRNA secondary structure detected using the dimethyl sulfate (DMS)-based method performed in a previous study^31^ and the SHAPE-MaP protocol adapted in this study. Nucleotides 32–518 of the 18S rRNA phylogenetic secondary structure^73^ are shown in the model and are colour-coded with SHAPE reactivities generated in this study. **c**, Pearson correlation among the four SHAPE-MaP biological replicates (by transcript) under each treatment condition. Nucleotides in 2,488 transcripts with read depth > 4,000 in all the replicates under all the conditions were used for the analysis. Boxes, IQR. Centre lines, median. Whiskers, values within 1.5 × IQR of the top and bottom quartiles. Circles represent Pearson correlation values for outliers. **d**, Cumulative fraction on the mutation rates of four nucleotides under each treatment condition.

Beyond primary sequences, we next considered a possible involvement of RNA secondary structures in this dynamic selection of translation start codons. To probe in vivo RNA secondary structural dynamics, we adapted selective 2′-hydroxyl acylation analysed by primer extension and mutational profiling (SHAPE-MaP) to detect global in planta changes in RNA secondary structure at nucleotide resolution with and without immune induction. This strategy relies on SHAPE reagents (here, 2-methylnicotinic acid imidazolide, NAI)—a group of hydroxyl-selective electrophiles that react with the 2′-hydroxyl position of unpaired residues of RNA^30^. The resulting 2′-O-adducts cause mutations in the cDNA during reverse transcription, which are detected through sequencing to create SHAPE reactivity profiles, yielding quantitative measurements of RNA structures inside the cell (Extended Data Fig. 3a). Regions with higher SHAPE reactivities are likely to be more single-stranded. To validate our protocol, we performed a targeted in planta SHAPE-MaP analysis of the *Arabidopsis* 18S rRNA. The signal obtained was consistent and significantly improved from that reported previously^31^ (Extended Data Fig. 3b).

**Extended Data Fig. 4:**
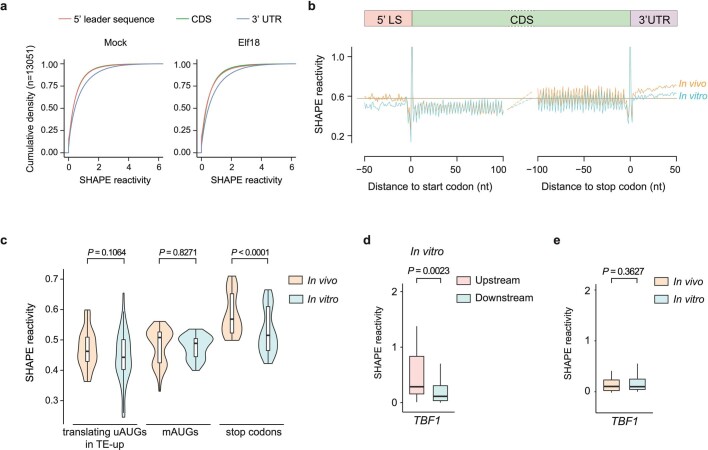
In vivo and in vitro SHAPE-MaP analyses depict RNA structural features. **a**, Cumulative fraction of the SHAPE reactivities of nucleotides in the 5′ leader sequence, CDS and 3′ UTR in mock- and elf18-treated samples. **b**, Average in vivo and in vitro SHAPE reactivities in the 5′ leader sequence (5′ LS), CDS and 3′ UTR across all expressed transcripts in the mock-treated samples aligned by the start and stop codons of CDS. Brown horizontal line marks the average in vivo SHAPE reactivity across all the nucleotides in mock-treated samples. **c**, Violin plots show the comparisons of in vivo and in vitro SHAPE reactivities of the 50 nt downstream regions of translating uAUGs in the TE-up transcripts and mAUGs in all expressed transcripts, as well as the 50 nt upstream region of stop codons in all expressed transcripts under the mock condition. **d**, Box plot shows the difference in in vitro SHAPE reactivities in the 50 nt upstream and the 50 nt downstream of uAUG2 in the *TBF1* transcript. **e**, Box plot shows the comparison of in vivo and in vitro SHAPE reactivities of the uAUG2-ds region in the *TBF1* transcript. For **c**–**e**, boxes represent IQR, centre lines mark median and whiskers indicate values within 1.5 × IQR of the top and bottom quartiles. *P* values were analysed by two-tailed Mann–Whitney tests.

We then performed the global in planta SHAPE-MaP analysis of mRNAs in *Arabidopsis* seedlings in response to mock treatment or treatment with elf18, which resulted in high-quality data (Extended Data Fig. 3c,d). To ensure accurate structure modelling, only data that passed the stringent cut-offs for read depth and completeness were used for subsequent analyses^30^ (Methods). We observed that, although the overall SHAPE reactivities of the 5′ leader sequences and coding sequences (CDSs) were comparable (Extended Data Fig. 4a), the nucleotides immediately downstream of the mAUGs in all expressed transcripts exhibited noticeably lower SHAPE reactivities, with the lowest values observed around +100 nucleotides (nt) (Fig. 2b), suggesting higher levels of double-stranded structures, protein binding or both in this region. We wondered whether this feature was related to start-codon recognition and translation initiation from mAUGs, and whether a similar feature exists for uAUGs. To answer these questions, we first examined the SHAPE reactivity for each of the 50 nt upstream and downstream of AUGs to determine whether there was a statistically significant difference. We found that nucleotides downstream of mAUGs and translating uAUGs exhibited significantly lower SHAPE reactivities compared to those upstream, but this was not observed for non-translating uAUGs (Fig. 2c). We further investigated whether the observed feature might contribute to the dynamic regulation of uAUG-mediated translation in the TE-up, TE-nc and TE-down transcripts (Fig. 1c,d). We found that, in the mock condition, translating uAUGs in the TE-up transcripts had significantly lower SHAPE reactivities in their downstream regions compared to those in the TE-nc and TE-down transcripts (Fig. 2c), with four TE-up transcripts shown in Fig. 2d.

To assess the possibility that the low SHAPE reactivity that was found downstream of mAUGs and translating uAUGs was a result of association with ribosomes or RNA-binding proteins, we performed global in vitro SHAPE-MaP experiments on the same samples in the mock condition. The overall SHAPE reactivities in vitro were lower than those observed in vivo, suggesting a lower degree of single-strandedness in vitro (Extended Data Fig. 4b), in line with previous findings^31–35^. Of note, we found that in the absence of proteins, the overall SHAPE reactivities in regions immediately downstream of mAUGs and translating uAUGs in the TE-up transcripts were not significantly changed from those obtained from the in vivo SHAPE-MaP (Extended Data Fig. 4b,c), indicating that the low SHAPE reactivities observed in this region are unlikely to be due to protein binding, but are more likely to be attributed to double-stranded RNA (dsRNA) secondary structures. Hence, we named these structures downstream of mAUGs and uAUGs ‘mAUG-ds’ and ‘uAUG-ds’, respectively. Targeted in vitro SHAPE-MaP analysis of the TE-up marker transcript *TBF1* also showed that the removal of proteins had no significant effect on the SHAPE reactivity patterns in its uAUG2-ds region (Extended Data Fig. 4d,e).

## Deep learning characterization of AUG-ds

**Extended Data Fig. 5:**
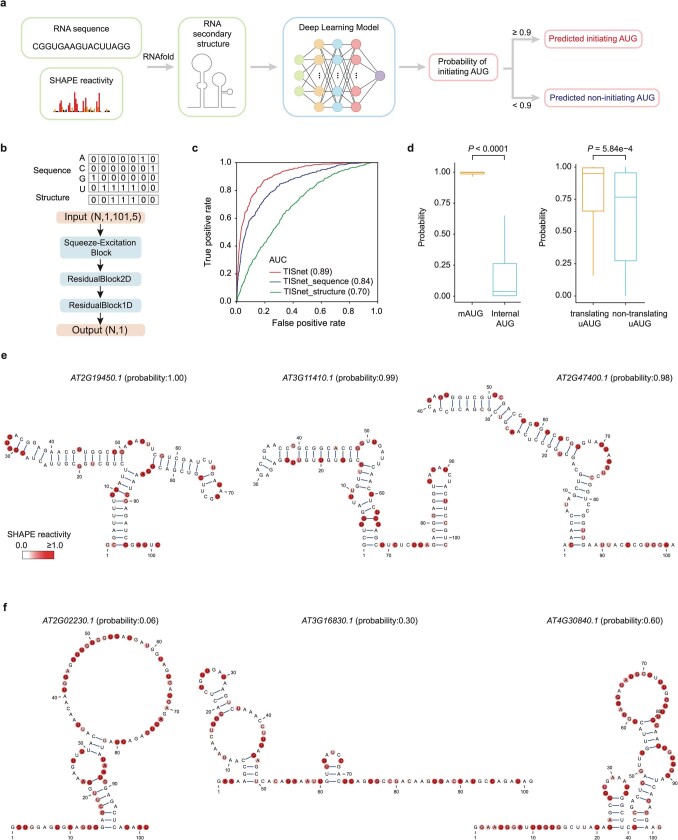
Deep learning analysis of the SHAPE-MaP data suggests that downstream double-stranded structures have a role in dictating AUG selection for translation initiation. **a**, Flow chart of TISnet. The RNA secondary structures downstream of AUGs were predicted by RNAfold^70^ constrained by SHAPE reactivities. TISnet predicted the probability of initiating AUG by integrating the RNA primary sequence and secondary structure information. AUGs with probability ≥ 0.9 are defined as predicted initiating AUGs, and AUGs with probability < 0.9 are defined as predicted non-initiating AUGs. **b**, The input data and architecture of TISnet. The input data of TISnet include RNA sequences encoded by one-hot encoding, and secondary structures encoded to 0 or 1. The TISnet architecture includes squeeze-excitation block, residual block (2D) and residual block (1D) adapted by the PrismNet model^69^. **c**, The receiver operating characteristic (ROC) curves of the TISnet models trained with both the sequence and the structure information (red line), or solely with the sequence information (blue line), or solely with the structure information (green line). The AUC (area under the ROC curve) scores of three models are shown. **d**, Box plot of the overall probabilities predicted by the TISnet model using downstream regions of mAUGs and internal AUGs (left) or translating and non-translating uAUGs (right). Boxes, IQR. Centre lines, median. Whiskers, values within 1.5 × IQR of the top and bottom quartiles. *P* values were analysed by two-tailed Mann–Whitney tests. Number of AUGs for the analysis: mAUGs, *n* = 2,857; internal AUGs, *n* = 7,143; translating uAUGs, *n* = 712; non-translating uAUGs, *n* = 314 (normalized read counts at these uAUGs = 0). **e**,**f**, Examples of RNA structural models of downstream regions of predicted initiating AUGs (**e**) and non-initiating AUGs (**f**).

To independently demonstrate that the observed structural patterns contribute to translation initiation from AUGs, we developed translation initiation site prediction using deep neural network (TISnet), based on the primary sequence, the structural data or both, to predict translation initiation sites. To train the TISnet model, data from mAUGs with high translational activities and internal AUGs were used as positive and negative samples, respectively (Methods). AUGs with a high probability (0.9 or higher) were classified as predicted initiating AUGs (Extended Data Fig. 5a,b). We found that the model achieved its best prediction performance—as shown by the high area under the receiver operating characteristic curve (AUC) score of 0.89 (Extended Data Fig. 5c)—only when both the sequence and the structural information were considered. There were clear differences in the predicted probabilities between mAUGs and internal AUGs (training data) and between translating uAUGs and non-translating uAUGs (testing data) (Extended Data Fig. 5d).

**Extended Data Fig. 6:**
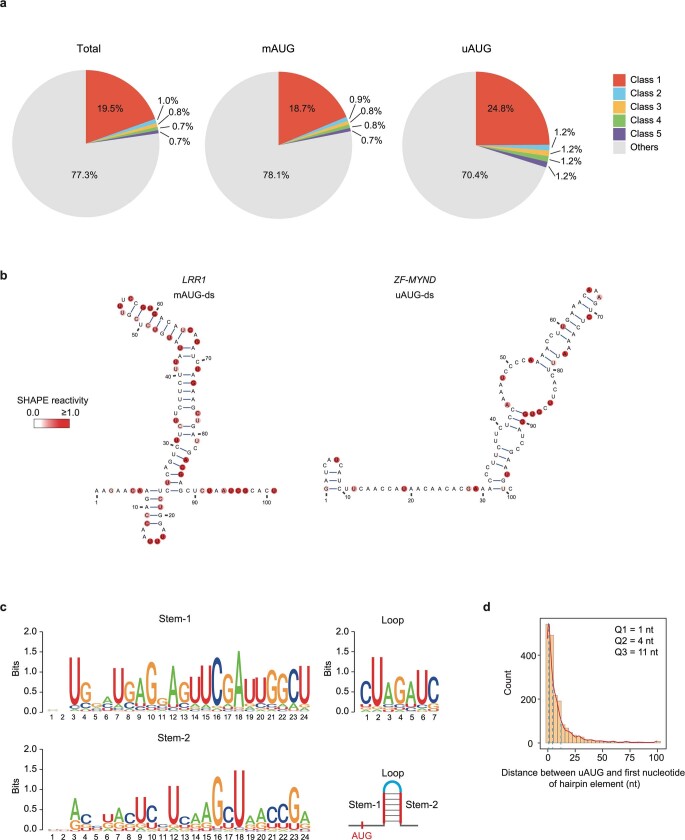
Characterization of class 1 AUG-ds. **a**, Pie plots show the percentage of different AUG-ds classes located in downstream regions of total predicted initiating AUGs (left), mAUGs in total predicted initiating AUGs (middle) and translating uAUGs in total predicted initiating AUGs (right). Each class of elements are defined by a group of hairpin elements with similar sequence patterns (see Methods for details). **b**, The secondary structure models of mAUG-ds in the *LRR1* transcript and uAUG2-ds in the *ZF-MYND* transcript in class 1. **c**, The position weight matrix (PWM) of the sequence motif of two stems and loop of the class 1 AUG-ds. **d**, Distribution of the distance between uAUG and the first nucleotide of the downstream hairpin element. Blue dashed lines represent the bottom (Q1), middle (Q2) and top (Q3) quartiles.

Our model further supports the hypothesis that mAUG-ds and uAUG-ds are responsible for the start-codon selection, because downstream regions of predicted initiating AUGs had significantly more negative folding energy than did predicted non-initiating AUGs (Fig 2e and Extended Data Fig. 5e,f). Most of the mAUG-ds and uAUG-ds exhibited a folding energy ranging from –19.9 kcal mol^–1^ to –34.1 kcal mol^–1^ and had 12 to 20 base pairs in the stem (Fig. 2f), with the nucleotide GC pair significantly enriched in the stem and UCU and UUC in the loop compared to the background (Fig. 2g). Hierarchical clustering on these elements according to the sequence similarities within loops and stems showed that the largest class (class 1) contains mAUG-ds and uAUG-ds in 341 out of 1,746 transcripts (19.5%), including *TBF1*, *ERECTA*, *LRR1* and *ZF-MYND* (Fig. 2h and Extended Data Fig. 6a–c). Moreover, most of the double-stranded structures begin within 25 nt downstream of uAUGs (Extended Data Fig. 6d). We next examined ribosomal occupancy on the predicted initiating uAUGs and non-initiating uAUGs and found a significant higher ribosome occupancy on the former than on the latter (Fig. 2i), suggesting that TISnet can also be used to accurately identify potential initiating uAUGs that have translational activities.

The pervasive presence of uAUG-ds in the TE-up transcripts is likely to contribute to the translation inhibitory roles of uAUGs under normal conditions, because downstream structures could slow the scanning of the translation preinitiation complex to enhance the chance of whole ribosome assembly^36,37^ and initiate translation from uAUGs instead of mAUGs. It is worth emphasizing that, in contrast to dsRNA structures upstream of AUGs, which normally inhibit translation^38^, the dsRNA structures downstream of AUGs identified in our study promote translation initiation.

## uAUG-ds dynamics in plants and human cells

**Fig. 3:**
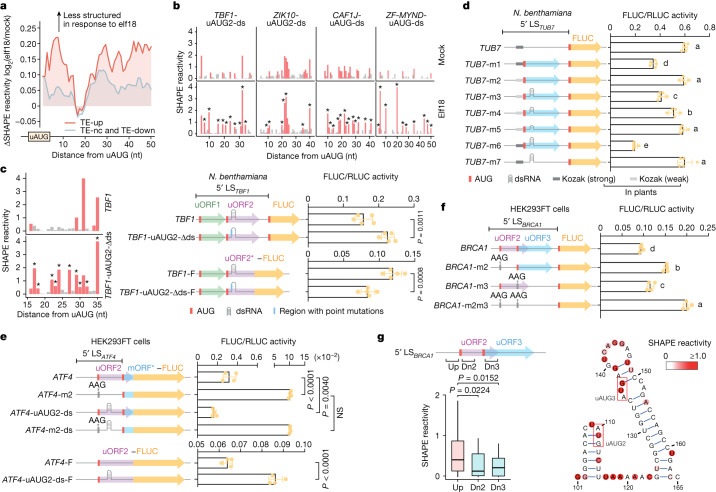
RNA secondary structures downstream of uAUGs dynamically regulate translation. **a**, elf18-induced average changes in SHAPE reactivity downstream of translating uAUGs in TE-up (red) or TE-nc and TE-down (grey) transcripts. **b**, elf18-induced changes in SHAPE reactivity across nucleotides downstream of major inhibitory translating uAUGs of four TE-up transcripts. Red bars, nucleotides with median-to-high SHAPE reactivities. Blue asterisks, elf18-induced increases in SHAPE reactivities. **c**, In vivo SHAPE-MaP probing of *TBF1* and *TBF1*-uAUG2-Δds (left) and dual-luciferase assay on their activities in controlling FLUC translation (right). 5′ LS_*TBF1*_, *TBF1* 5′ leader sequence. *TBF1*-F and *TBF1*-uAUG2-Δds-F, FLUC fused in-frame with the first 66 nt of uORF2 (uORF2*). **d**, Addition of uAUG and/or dsRNA structures affects synthetic reporter translation. All reporters have the same 5′ leader sequence length but different folding energies in the downstream region (100 nt) of uAUG: *TUB7*, *TUB7*-m1 and *TUB7*-m2 (−9.8 kcal mol^−1^); *TUB7*-m5 (−14.3 kcal mol^−1^); *TUB7*-m4 (−16.9 kcal mol^−1^); *TUB7*-m3, *TUB7*-m6 and *TUB7*-m7 (−23.6 kcal mol^−1^). **e**, Addition of an artificial hairpin downstream of uAUG2 further inhibits mammalian ATF4 translation. mORF*-FLUC, FLUC fused in-frame with the first 84 nt of *ATF4* mORF. *ATF4*-m2, uAUG2 mutated to AAG. *ATF4*-uAUG2-ds, the downstream region of uAUG2 substituted with a hairpin without changing its length. *ATF4*-m2-ds, *ATF4*-uAUG2-ds with uAUG2 mutated to AAG. *ATF4*-F and *ATF4*-uAUG2-ds-F, FLUC fused in-frame with uORF2. **f**,**g**, Translation of mammalian *BRCA1* is regulated by uAUGs (**f**) and their downstream dsRNA structures detected by in vivo SHAPE-MaP (**g**). Boxes, IQR; centre lines, median; whiskers, values within 1.5 × IQR of the top and bottom quartiles. *P* values for **c**–**f**, two-tailed Student’s *t*-test; for **g**, two-tailed Mann–Whitney tests. Values are mean ± s.d. (*n* = 5 biological replicates in **c**,**d**; *n* = 4 biological replicates in **e**,**f**). For **d**,**f**, different letters indicate statistically significant differences (*P* < 0.05).

Because our Ribo-seq data revealed an elf18-triggered shift in translation from uORFs to mORFs in the TE-up transcripts (Fig. 1c,d), we hypothesized that this global translational reprogramming is regulated by structural changes of uAUG-ds. Indeed, we observed an overall elf18-induced increase in SHAPE reactivities in the uAUG downstream regions (Fig. 3a), suggesting a general enhancement in the unwinding of these regions in response to immune induction. More importantly, the extent of the change is much bigger in the TE-up transcripts than in the TE-nc and TE-down transcripts (Fig. 3a), highlighting the greater effect of immune induction on the structural changes of uAUG-ds in the TE-up transcripts. Closer examination of the four TE-up transcripts confirmed our global observation (Fig. 3b). We propose that the immune-induced reduction in uAUG-ds structural complexity allows the preinitiation complex to scan beyond the uAUGs to initiate translation from downstream mAUGs.

**Extended Data Fig. 7:**
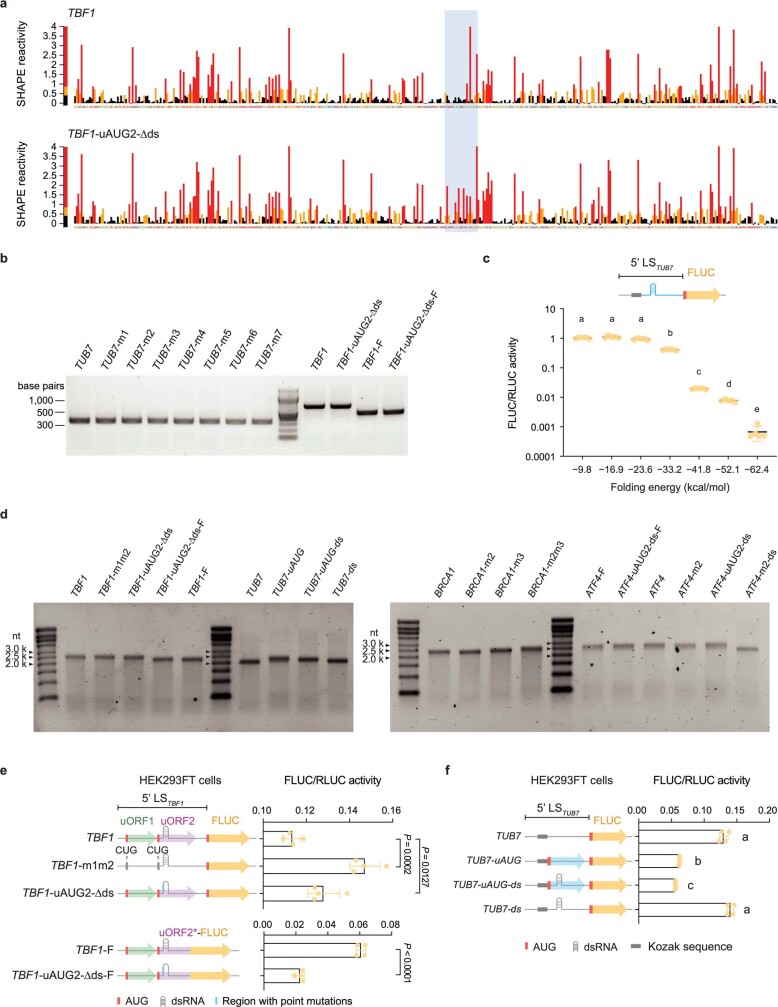
uAUG-ds dynamically regulates translation in plants and mammalian cells. **a**, Overview of in vivo SHAPE reactivities across the 5′ leader sequences of *TBF1* (top) and *TBF1-uAUG2-Δds* (bottom) expressed in *N. benthamiana*. The mutated uAUG-ds region is highlighted in blue. **b**, DNA gel electrophoresis showing the 5′ RACE results of *TBF1*, *TUB7* and their mutation variants (corresponding to Fig. 3c,d). **c**, Effects of different strengths of dsRNA structures on the translation of the synthetic reporter (no uAUG). The dsRNA structures were introduced without changing the length of 5′ leader sequences. Folding energies were calculated for the region (blue) 54–153 nt downstream of the 5′ end. 5′ LS_*TUB7*_, the 5′ leader sequence of *TUB7*. Data were analysed by two-tailed Student’s *t*-test. Different letters indicate statistically significant differences (*P* < 0.05). Values are mean ± s.d. (*n* = 5 independent biological replicates). **d**, In-vitro-transcribed RNAs used in transfecting HEK293FT cells (corresponding to Fig. 3e,f and Extended Data Fig. 7e,f). **e**, Translational regulatory activity of the *Arabidopsis TBF1* 5′ leader sequence (5′ LS_*TBF1*_) is maintained in HEK293FT cells. Mutagenesis of the 5′ leader sequence of *TBF1* showed that, in HEK293FT cells, as in *Arabidopsis*, the double-stranded structure downstream of uAUG2 is required for inhibiting the reporter translation (top) by enhancing translation initiation from uAUG2 (bottom). *TBF1*-F and *TBF1*-uAUG2-Δds-F are FLUC fused in-frame with the first 66 nt of uORF2 (uORF2*). *P* values were calculated by two-tailed Student’s *t*-test. Values are mean ± s.d. (*n* = 4 independent biological replicates). **f**, Effects of uAUG and RNA double-stranded structures on the synthetic reporter translation in HEK293FT cells. Data were analysed by two-tailed Student’s *t*-test. Values are mean ± s.d. (*n* = 4 independent biological replicates). In **c**,**e**,**f**, each dot represents a biological replicate.

To validate the role of uAUG-ds in dynamically dictating start-codon selection and thus regulating downstream protein production, we first examined the uAUG2-ds in the 5′ leader sequence of the *TBF1* transcript (*TBF1*-uAUG2-ds), using dual-luciferase reporters that were transiently expressed in *Nicotiana benthamiana*^22^. When we disrupted the base pairs in the hairpin structure by introducing point mutations in uAUG2-ds (*TBF1*-uAUG2-Δds) to mimic its structural opening in response to elf18 (Fig. 3c, left and Extended Data Fig. 7a,b), we observed a significant increase in the FLUC/RLUC activity (Fig. 3c, right). The role of uAUG2-ds in enhancing translation initiation from uAUG2 was further substantiated using another reporter in which FLUC is fused in-frame with uAUG2 instead of mAUG (Fig. 3c, right). Altogether, these results show that a double-stranded structure downstream of uAUGs (uAUG2 for the *TBF1* transcript), instead of specific protein binding, is conducive to the uORF-mediated inhibition of downstream mORF translation by facilitating translation initiation from the uAUGs. This inhibition may be alleviated during stress, when the RNA double-stranded structure is unwound to allow the translation preinitiation complex to scan beyond uAUGs to initiate mORF translation.

To show that the dynamic function of uAUG-ds in regulating translation initiation is generalizable, we engineered a reporter using the naive 5′ leader sequence of the *Arabidopsis TUB7* (tubulin beta-7) gene to drive the translation of FLUC in the dual-luciferase reporter system. We then mutagenized the 5′ leader sequence, without changing its length (Extended Data Fig. 7b), to introduce a uAUG in a strong or a weak Kozak context with or without artificial dsRNA structures (Fig. 3d). The resulting reporter activities showed that in addition to the Kozak sequence context, the uAUG-ds structures within the optimal range (that is, 12–20 base pairs; –19.9 to –34.1 kcal mol^–1^ in Fig. 2f) enhanced the recognition of uAUG for translation initiation and consequently dampened downstream reporter translation (Fig. 3d). However, in the absence of the uAUG, the structure alone did not inhibit downstream reporter translation (Fig. 3d, *TUB7*-m7), as long as it was within the optimal range of folding energy (Extended Data Fig. 7c), further supporting the role of uAUG-ds in engaging the ribosome to initiate translation from uAUGs.

To test whether the uAUG-ds-mediated translation initiation occurs in animals, we expressed the in-vitro-transcribed synthetic *TUB7* reporter mRNAs and the *Arabidopsis TBF1* reporter mRNAs in human HEK293FT cells (Extended Data Fig. 7d), and found that uORF-mediated reporter translation was most inhibited when uAUG-ds was present (Extended Data Fig. 7e,f). This result indicates that dsRNA enhances uAUG translation initiation in both plants and a human cell line. This conclusion was further supported when we introduced a dsRNA structure downstream of the uAUG2 in in-vitro-transcribed *ATF4*, a well-known mammalian stress-responsive gene^13^. This further inhibited the translation of *ATF4* through enhanced translation initiation from the uAUG2 (Fig. 3e and Extended Data Fig. 7d).

We then showed that uAUG-ds structures are present in mammalian transcripts, by performing in vivo SHAPE-MaP analysis on a mutant version of the tumour suppressor *BRCA1* mRNA that is found in breast cancer tissue. The translation of this mutant *BRCA1* mRNA is known to be inhibited by uAUG2 and uAUG3, with uAUG2 having a stronger inhibitory effect than uAUG3^39^ (Fig. 3f). Significantly lower SHAPE reactivities were detected downstream of uAUG2 and uAUG3, as compared with their upstream regions (Fig. 3g, left), further supporting our claim that uAUG-ds (Fig. 3g, right), instead of a primary protein-binding sequence, could be a universal mechanism for dynamic start-codon selection for translation initiation.

## Immune-induced helicases unwind uAUG-ds

**Fig. 4:**
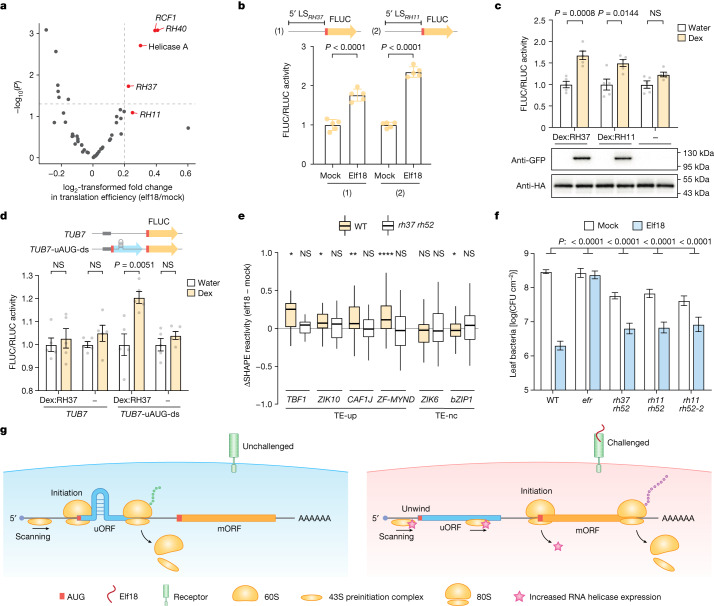
RNA helicases unwind RNA secondary structures downstream of uAUGs to alleviate repression of mAUG translation. **a**, Volcano plot of changes in translational efficiency for 54 known *Arabidopsis* RNA helicases after treatment with elf18. **b**, Translational responses of the 5′ leader sequences of *RH37* (5′ LS_*RH37*_) and *RH11* (5′ LS_*RH11*_) to elf18. *P* values were calculated by two-tailed Student’s *t*-test. Values are mean ± s.d. (*n* = 5 independent biological replicates). **c**, Effect of dex-induced expression of YFP-tagged RNA helicases (RH37 and RH11) (bottom) on translation of the *35S:TBF1 5*′ *LS-FLUC/35S:RLUC* dual-luciferase reporter (top). HA-tagged RLUC levels were detected as internal controls. **d**, Effect of dex-induced expression of YFP-tagged RH37 on translation of the *TUB7* synthetic reporters (top). For **c**,**d**, *P* values were calculated by two-tailed Student’s *t*-test. Values are mean ± s.e.m. (*n* = 5 independent biological replicates). **e**, Box plots of in planta changes in SHAPE reactivity in the endogenous uAUG-ds regions of four TE-up and two TE-nc transcripts in wild-type (WT) and the helicase-mutant (*rh37* *rh52*) plants. For transcripts with two translating uAUGs (*TBF1*, *ZIK10*, *ZIK6* and *bZIP1*), changes in the downstream region of the major inhibitory uAUGs (that is, uAUG2-ds) are shown. Data were analysed by two-tailed Wilcoxon signed-rank tests. *P < 0.05, **P < 0.01, ****P < 0.0001. Boxes, IQR; centre lines, median; whiskers, values within 1.5 × IQR of the top and bottom quartiles. **f**, Elf18-induced protection against *Psm* ES4326 in wild-type plants and helicase mutants (*n* = 12 plants). Bacterial growth (in colony-forming units; CFU) was measured two days after inoculation and is shown as mean ± s.e.m. *P* values were calculated by two-way ANOVA. The experiment was repeated twice with similar results. **g**, A model of RNA-secondary-structure-mediated translational regulation of uORF-containing transcripts during PTI.

**Extended Data Fig. 8:**
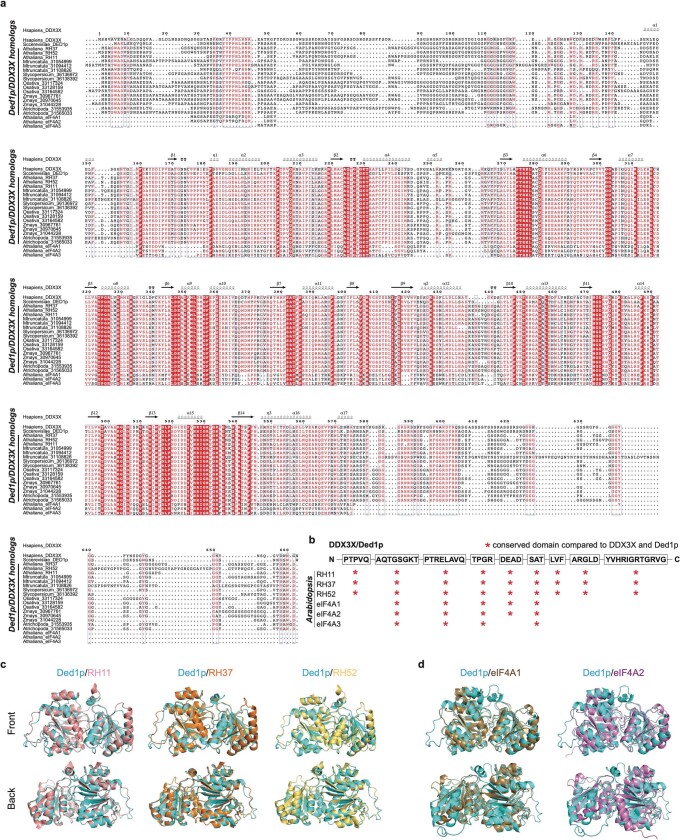
Structural similarities of *Arabidopsis* RNA helicases RH11, RH37 and RH52 to yeast Ded1p and mammalian DDX3X. **a**, Protein sequence alignment of *Arabidopsis* RH11, RH37 and RH52 with their homologues in five other angiosperm species: *Amborella trichopoda* (Atrichopoda), *Zea mays* (Zmays), *Oryza sativa* (Osativa), *Solanum lycopersicum* (Slycopersicum), *Medicago truncatula* (Mtruncatula), together with yeast Ded1p, human DDX3X and *Arabidopsis* eIF4A homologues. Numbers following each name are PACIDs. ESPript 3.0 (ref. ^74^) was used for visualization of protein sequence alignment. Human DDX3X structure elements^75^ were used as references. **b**, Domain conservation of *Arabidopsis* RH11, RH37, RH52, eIF4A1, eIF4A2 and eIF4A3 with DDX3X/Ded1p regarding the nine sequence motifs (in the boxes and illustrated from N terminus to C terminus). Conserved domains are indicated with red asterisks. **c**,**d**, Pairwise alignment of yeast Ded1p with *Arabidopsis* RH11, RH37 and RH52 (**c**) and with *Arabidopsis* eIF4A1 and eIF4A2 (**d**) shows that RH11, RH37 and RH52, but not eIF4A1 and eIF4A2, are structurally similar to Ded1p. Protein structures were predicted by AlphaFold^44^, and superimposed and visualized by PyMol v.1.3.

We next sought to answer the question of how uAUG-ds is unwound to facilitate immune-induced translation in plants. Previous studies have suggested that some DEAD-box RNA helicases can serve as alternatives to the canonical eukaryotic translation initiation factor 4A (eIF4A) in the preinitiation complex to unwind RNA for translation^40–42^. To identify potential candidates for the elf18-induced unwinding of uAUG-ds, we examined the changes in translational efficiency of the 54 known RNA helicases in *Arabidopsis*, and found 4 candidates that showed significant translational induction in response to elf18 (Fig. 4a). Among them, only RH37 was predicted to be localized in the cytoplasm. A genome-wide homology analysis across angiosperms revealed another two close RH37 homologues, RH11 and RH52 (Extended Data Fig. 8a), consistent with another study^43^. The translational inducibility by treatment with elf18 was confirmed for RH37 and RH11 using the dual-luciferase assay, in which the 5′ leader sequences of these helicase transcripts were used to drive the FLUC translation (Fig. 4b). Moreover, through comparisons of protein amino acid sequences, functional domains and structures predicted by AlphaFold^44^, we found that RH11, RH37, and RH52 are orthologous to the yeast Ded1p and human DDX3X (Extended Data Fig. 8b–d). The sequence and structural homology to the yeast Ded1p also aligns well with the anticipated function for RH11, RH37 and RH52, because the yeast Ded1p, which functions with other translation initiation factors in the preinitiation complex, is required to unwind highly structured regions in 5′ leader sequences during translation initiation^42,45^. Consistently, a previous study revealed that the *Arabidopsis* RH11 interacts with translation initiation factors^22^. In addition, mutating the yeast Ded1p helicase causes enhanced translation initiation from near-cognate start codons upstream of structured regions^46^. We hypothesized that, opposite to the helicase mutant, immune-induced increases in the levels of RH11, RH37 and RH52 might promote the unwinding of uAUG-ds, thus alleviating the uAUG-mediated inhibition of mORF translation.

To test our hypothesis, we built the constructs *Dex:RH37-YFP* and *Dex:RH11-YFP* to put the transcription of *RH37-YFP* and *RH11-YFP* under the control of a dexamethasone (dex)-inducible system^47^ and transiently coexpressed each with the dual-luciferase reporter driven by the 5′ leader sequence of *TBF1* in *N. benthamiana*. Notably, we observed a significant increase in the FLUC activities four hours after treatment with dex (Fig. 4c). This suggests that a transient increase in the expression of these RNA helicases could lead to enhanced translation of *TBF1*.

We next showed that the effect of these helicases is through remodelling of uAUG-ds, because it was only observed when RH37 was coexpressed with the synthetic *TUB7* reporter that contains uAUG-ds (Fig. 4d). This result once again demonstrates that uAUG-ds can serve as a molecular switch to dynamically regulate translation initiation.

**Extended Data Fig. 9:**
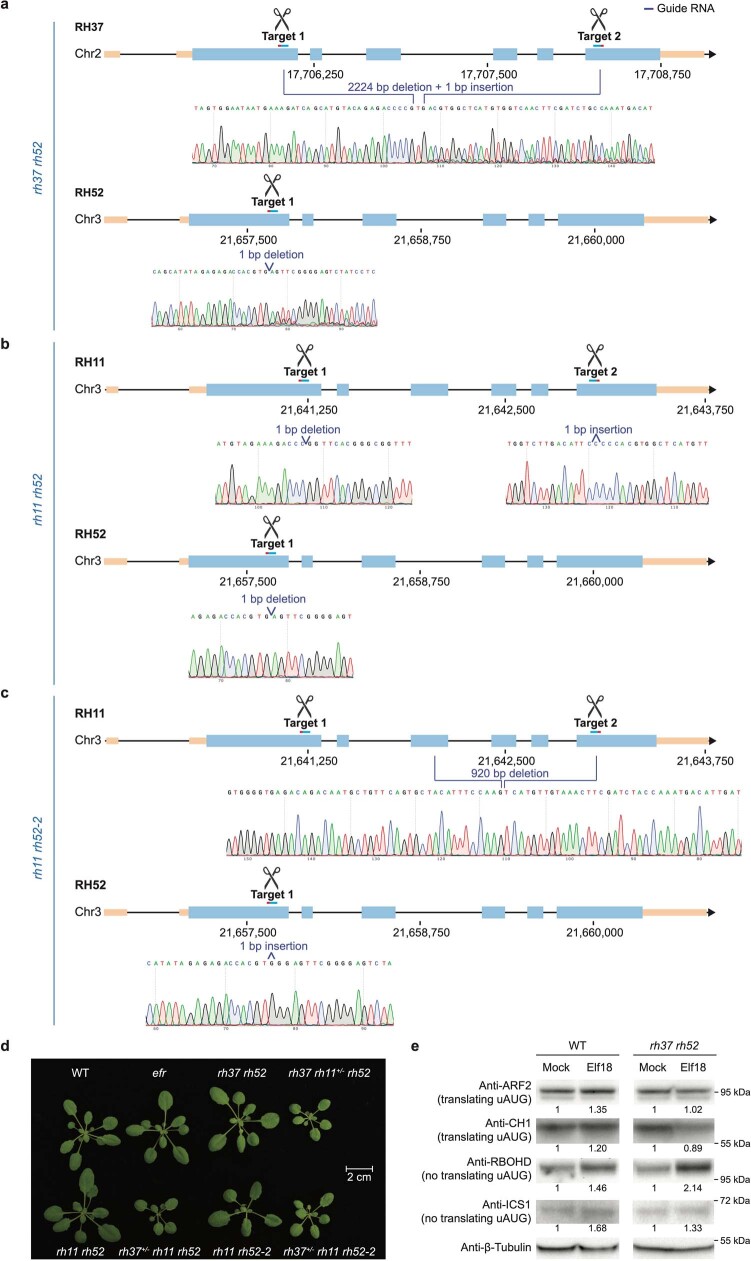
Genotyping and phenotypes of the helicase mutants. **a**–**c**, Schematics of CRISPR experiments and the Sanger sequencing results from *rh37 rh52* (**a**), *rh11 rh52* (**b**) and *rh11 rh52-2* (**c**) double mutants. Short blue line, guide RNA; red dot at the end of the short blue line, PAM sequence. **d**, Representative morphology of WT, *efr*, *rh37 rh52*, *rh11 rh52* and *rh11 rh52-2* plants before the elf18-induced protection assay. Higher-order mutants *rh37 rh11*^*+/*–^ *rh52*, *rh37*^*+/*–^ *rh11 rh52*, and *rh37*^*+/*–^ *rh11 rh52-2* are included in the photo to show their growth defect. **e**, Western blotting shows that the helicase double mutant (*rh37 rh52*) specifically compromises the elf18-mediated increases in protein levels from translating uAUG-containing transcripts (*ARF2* and *CH1*), but not from transcripts without translating uAUGs (*RBOHD* and *ICS1*). The relative band intensity of the immunoblot (represented by numbers below the blot) was normalized to mock for each background. The experiment was repeated twice with similar results.

Finally, to confirm the roles of the RH11, RH37 and RH52 helicases in elf18-induced translation genetically, we generated *rh37* *rh52*, *rh11* *rh52*, and *rh11* *rh37* double-mutant lines using a high-efficiency CRISPR method^48^ (Extended Data Fig. 9a–c). Because the *rh11* *rh37* mutant exhibited a developmental defect, whereas the *rh37* *rh52* and the *rh11* *rh52* plants had almost wild-type morphology (Extended Data Fig. 9d), we chose to use *rh37* *rh52* for targeted in planta SHAPE-MaP of the endogenous transcripts representing the TE-up and TE-nc groups. We found that in the TE-up transcripts, the elf18-induced structural opening in the downstream regions of uAUGs was observed in wild-type plants, but diminished in the helicase double mutant (Fig. 4e), supporting the involvement of RH11, RH37 and RH52 in elf18-induced unwinding of uAUG-ds. Moreover, we examined elf18-induced changes in the levels of four proteins with available antibodies in wild-type and helicase-mutant (*rh37* *rh52*) plants and showed that increases in protein levels from those two transcripts containing translating uAUGs were dependent on the helicase activities (Extended Data Fig. 9e).

To examine the global effect of the helicase mutations on elf18-induced resistance against pathogens, we performed bacterial infection using *Pseudomonas syringue* pv*. maculicola* ES4326 (*Psm* ES4326) in wild-type, *rh37* *rh52* and *rh11* *rh52* mutant plants after pre-treating plants with elf18. As a negative control, we included the elf18 receptor mutant, *efr*. We found that the helicase mutants had higher basal resistance to *Psm* ES4326 than did the wild-type plants (Fig. 4f), suggesting that they might affect transcripts other than those involved in PTI. Nevertheless, the helicase mutants had significantly diminished sensitivity to elf18-induced resistance, resulting in more overall bacterial growth (Fig. 4f); this clearly shows that these helicases have indispensable roles in the translational regulation of PTI. Altogether, our results show that the elf18-inducible RNA helicase RH37 and its homologues RH11 and RH52 are involved in unwinding uAUG-ds in the TE-up transcripts and in promoting the translation of downstream defence proteins against pathogen challenges.

## Discussion

In this study we have discovered that uAUG-ds is crucial for dynamic start-codon selection for translation initiation during plant PTI. Without stress, the translation of defence proteins is inhibited by uORFs, owing to the presence of uAUG-ds, which slows the scanning of the preinitiation complex to engage the ribosome to initiate translation from uAUGs instead mAUGs. In response to stress, the expression of RH37-like helicases, known to be associated with the translation preinitiation complex^22,42,45,46^, is increased to facilitate the unwinding of uAUG-ds, thus promoting the bypass of uAUGs and the translation of downstream defence proteins (Fig. 4g).

**Extended Data Fig. 10:**
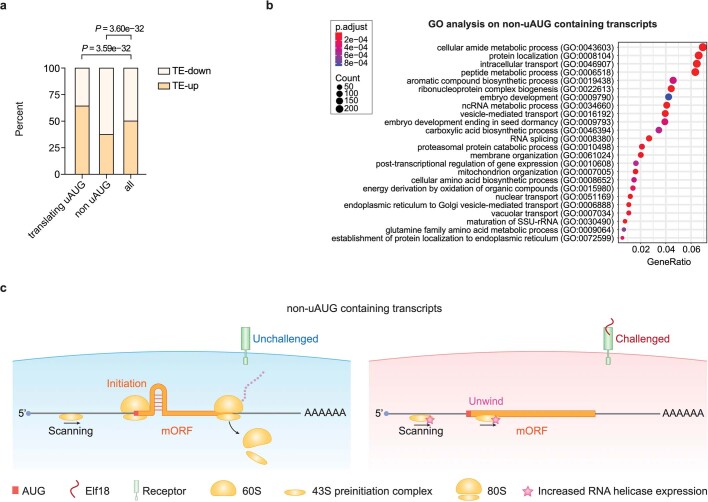
Proposed mechanism for translational regulation of non-uAUG-containing transcripts. **a**, Percentage comparison of translating uAUG-containing, non-uAUG-containing and all transcripts with increased or decreased translation efficiency after elf18 induction (TE-up or TE-down). TE-up: transcripts with upregulated TE (*P* value < 0.05, log_2_-transformed fold change > 0.16); TE-down: transcripts with downregulated TE (*P* value < 0.05, log_2_-transformed fold change < –0.16). **b**, GO enrichment analysis on the non-uAUG-containing transcripts. **c**, A proposed model of mAUG-ds-mediated translational regulation of non-uAUG-containing transcripts during PTI.

Although this study was initiated to study uAUG-modulated translation in a plant immune response, the pervasive presence of the dsRNA structures downstream of both mAUGs and translating uAUGs (Fig. 2b,c), the unbiased deep learning results (Fig. 2e–i and Extended Data Figs. 5 and 6), and the functional data obtained from studies in both plants and mammalian systems (Fig. 3 and Extended Data Fig. 7) strongly support the fundamental importance of mAUG-ds and uAUG-ds in regulating translation in general. In contrast to the Kozak sequence context, which is crucial for start-codon recognition in static conditions, the uAUG-ds discovered in this study can be dynamically remodelled in response to stimuli to reprogram translation. Notably, such dynamic regulation also occurs for transcripts that contain only mAUG-ds, which are enriched with transcripts in the TE-down category in response to elf18 treatment and found to encode growth-related proteins (Extended Data Fig. 10a,b). This finding indicates that immune-induced helicases can also unwind mAUG-ds and reduce translation from mAUG to inhibit the production of growth-related proteins (Extended Data Fig. 10c).

Our discovery of AUG-ds in this study was only possible through the integrated application of transcriptome-wide translational and structural analyses and deep learning algorithms, because such structural features are unlikely to be detected through sequence homology. The strategy used here can be readily expanded to identify and characterize AUG-ds structures in other organisms, as AUG-ds regulate translation in different organisms (Fig. 3 and Extended Data Fig. 7). Indeed, the observed global structural patterns of mRNA surrounding mAUGs in yeast^49^ and *Caenorhabditis elegans*^50^ are consistent with the presence of mAUG-ds. Together with the fact that Ded1p, DDX3X and RH37 helicases are highly conserved from plants to humans (Extended Data Fig. 8), we hypothesize that the uAUG-ds–RNA helicase regulatory module is broadly present in eukaryotes. Moreover, the general features of mAUG-ds and uAUG-ds revealed in this study (Fig. 2e–g) provide information for the rational design of protein synthesis for basic research as well as for applications in agriculture, in medicine and beyond. Using well-trained deep learning models in different organisms, potential uAUG-ds of functional genes can be identified to manipulate their translation. Our success in engineering an inducible translational reporter that functions in plants as well as in human cells (Figs. 3d and 4d and Extended Data Fig. 7f) gives us confidence in the applicability of uAUG-ds as a molecular switch for regulating gene expression.

## Methods

### Plant growth, treatment with elf18 and transformation

*Arabidopsis* seedlings were grown on 1/2 Murashige and Skoog (MS) plates containing 0.8% agar and 1% sucrose or in soil, both at 22 °C under 12–12-h light–dark cycles with 55% relative humidity. Unless specified, all *Arabidopsis* plants used in the experiments were in the Col-0 background. *N. benthamiana* plants were grown under the same conditions in soil as those for *Arabidopsis* for four to five weeks before experiments. For treatment with elf18, *Arabidopsis* seedlings were grown on plates for seven days, transferred to liquid 1/2 MS solution and grown for one more day before being treated with 10 μM elf18 or water for 1 h. Transgenic plants were generated using the agrobacterium-mediated transformation method involving floral dipping^51^.

### Cell line

The HEK293FT cell line was purchased from the Duke Cell Culture Facility (Invitrogen, R700-07). All cells tested negative for mycoplasma contamination. Cell line identity was confirmed by STR authentication. Cells were cultured in Dulbecco’s modified Eagle’s medium (DMEM) supplemented with 10% heat-inactivated fetal bovine serum and 100 U ml^−1^ penicillin-streptomycin at 37 °C and incubated with 5% CO_2_, 95% air.

### Plasmid construction

The backbone (pTC090-32) for the dual-luciferase constructs used for expression in plants was generated in a previous study^22^. The 5′ leader sequences of the transcripts being tested were PCR-amplified from the Col-0 cDNA, and that of the *TUB7* transcript was synthesized by IDT before being inserted into the backbone through ligation-based reactions (NEB) or using the ClonExpress II One Step Cloning Kit (Vazyme). The site mutations and hairpin structures were introduced by primer-based PCR.

For in vitro transcription and expression in the mammalian cell line, the 5′ leader sequence of the *ATF4* transcript was PCR-amplified from the normal lung fibroblast cell line IMR90 cDNA. The 5′ leader sequence of the *BRCA1* transcript was PCR-amplified from genomic DNA from the human breast cancer cell line MCF7. All of the 5′ leader sequences were cloned into the plasmid backbone with the FLUC reporter by Gibson Assembly (NEB). The site mutations and hairpin structures were introduced by primer-based PCR.

To generate the plasmids with dex-inducible expression of RNA helicases, the CDSs of RH11 and RH37 were PCR-amplified from the Col-0 cDNA and cloned into pBSDONR p1-p4, separately. Each of these clones was then paired with the YFP tag, which was cloned in pBSDONR p4r-p2, to generate fusion constructs in the pBAV154 destination vector by multisite LR reaction (LR clonase II plus, Thermo Fisher Scientific). The CRISPR knock-out lines were built through a highly efficient multiplex editing method^48^. In brief, to construct the shuttle vectors, four guide RNA sequences, TAAACCGCCCGTGAACCACG, TAGACTCCCCGAACTCCACG, TAGACTGTTCGTGAACCACG and TGGTCTTGACATTCCCCACG, were loaded into the pDEG332, pDEG333, pDEG335 and pDEG337 modules, respectively. Then these guide RNA sequences were assembled into arrays in the recipient vector (pDGE666).

All of the primers and oligos used in this study are listed in Supplementary Table 1. All constructs were confirmed by Sanger sequencing before use.

### Ribo-seq and RNA sequencing

*Arabidopsis* seedlings treated with elf18 or water as described above were collected, frozen in liquid nitrogen and ground using the Genogrinder (SPEX SamplePrep). Polysome profiling was performed as described previously^20^. In brief, the ground tissue was homogenized in the polysome extraction buffer and centrifuged to remove cell debris. The supernatant was then layered on top of a sucrose cushion and the ribosome pellet was collected after ultracentrifugation. The pellet was then washed with cold water and subjected to RNase I (Ambion) digestion. The reaction was quenched by adding SUPERaseIn (Invitrogen). Ribosome-bound RNA was purified and subjected to treatment with PNK (NEB) and size selection through gel (Invitrogen) extraction. The recovered RNA was then subjected to library preparation using the NEBNext Multiplex Small RNA Library Prep Kit with slight modifications. Specifically, after the reverse transcription, rRNA depletion was performed. In brief, the cDNA product was cleaned up with the Oligo Clean & Concentrator Kit (Zymo) and then eluted with water. The eluted product was mixed with 0.4-nmol probes used in previous studies^20,52^ in the saline-sodium citrate (SSC) solution, and the mixture was subjected to denaturation at 100 °C for 90 s, followed by a gradual decrease of temperature from 100 °C to 37 °C to allow annealing of the ribosomal DNA (rDNA) and the biotinylated oligos. The mixture was then incubated with 200 μg pre-washed Dynabeads MyOne Streptavidin C1 beads (Invitrogen) for 15 min at 37 °C with constant shaking. The tube was then placed on a magnetic rack for another 5 min and the flow-through was collected and cleaned up using the Oligo Clean & Concentrator Kit (Zymo). This rDNA-depleted product was used as the template for PCR amplification and library preparation. The Agilent 2100 Bioanalyzer was used for the sample quality control (Extended Data Fig. 1a). RNA from the same lysate was isolated and subjected to library preparation using the KAPA Stranded mRNA-Seq Kit (Roche). The six libraries for Ribo-seq (three mock and three elf18-induced) were pooled at equal amounts of DNA and subjected to next-generation sequencing using the Illumina NovaSeq (S2, full flow cell) with paired-end reads of 50 bp in length. The six libraries for RNA sequencing (RNA-seq) (three mock and three elf18-induced) were pooled at equal amounts of DNA and subjected to next-generation sequencing using the Illumina NovaSeq (S Prime, 1 lane) with paired-end reads of 50 bp in length.

### Ribo-seq and RNA-seq data processing

Ribo-seq read processing was performed following the steps shown in Extended Data Fig. 2a. Specifically, raw reads were trimmed using Trim Galore v.0.6.6, a wrapper tool of Cutadapt^53^ and FastQC^54^. The trimmed reads with a length longer than or equal to 24 nt and shorter than or equal to 35 nt were kept and mapped to the rRNA and tRNA library from the *Arabidopsis* TAIR 10 genome using Bowtie 2 v.2.4.2 (ref. ^55^). The unmapped reads were then assigned to the *Arabidopsis* TAIR 10 genome using STAR v.2.7.8a (ref. ^56^) with –outFilterMismatchNmax 3 –outFilterMultimapNmax 20 –outSAMmultNmax 1 –outMultimapperOrder Random. FastQC v.0.11.9 (ref. ^54^) and MultiQC v.1.9 (ref. ^57^) were applied for quality control during each step. Similarly, RNA-seq reads were trimmed and mapped using the same programs under default parameters.

To assess the data quality, we first determined the read length distribution (Extended Data Fig. 1b) and the reads per kilobase of transcript per million mapped reads (RPKM) for all the transcripts in each replicate for the RNA-seq- and Ribo-seq-mapped reads using the featureCount program^58^ embedded in the Subread package v.2.0.3, and plotted the Pearson correlations between every two replicates (Extended Data Fig. 1c,d). Then we determined the P-site offset near start and stop codons for reads with a length ranging from 24 nt (24-mers) to 35 nt (35-mers) in Ribo-seq using Plastid v.0.6.1 (ref. ^59^; Extended Data Fig. 1e). Next, we determined the nucleotide periodicity 300 nt downstream of the start codons by calculating the power spectral density (Extended Data Fig. 1f). In addition, we calculated the distribution of RNA-seq and Ribo-seq reads in the 5′ leader sequence, CDS and 3′ UTR of each transcript from mock- and elf18-treated samples (Extended Data Fig. 1g). A metaplot of the normalized distribution of Ribo-seq reads on the normalized transcript was calculated using the computational genomics analysis toolkit (CGAT)^60^ (Extended Data Fig. 1h). Changes in translational efficiency were calculated using deltaTE^61^. GO enrichment was performed online using the Gene Ontology resource^23–25^ (http://geneontology.org/) and the results were visualized using enrichplot^62^.

### Identification of translating mAUGs and uAUGs

To identify transcripts with detectable translation initiation from mAUGs, we analysed 25,554 detected transcripts that had an RPKM of exon ≥ 1 in all of the six RNA-seq samples and a RPKM of CDS ≥ 1 in all of the six Ribo-seq samples (Extended Data Fig. 2a). We then calculated ribosome footprints spanning every mAUG for all the 25,554 detected transcripts and normalized each count by total read count and transcript abundance. To set the background read count, we took the top (Q3) quartile of the normalized read counts from regions 50 nt upstream of mAUGs of 5,482 transcripts that have 5′ leader sequences ≥ 100 nt without uAUGs (Extended Data Fig. 2b). Using the resulting background cut-off at 23.17, transcripts with normalized read counts at mAUG ≥ 23.17 and with raw read counts at mAUG ≥ 10 in all of the six Ribo-seq samples were retained, and this yielded 13,051 ‘expressed transcripts’ with detectable translation initiation from mAUGs (Extended Data Fig. 2a).

To identify the uAUGs that can engage ribosomes and facilitate translation initiation, we performed similar calculation and normalization steps for ribosome footprints spanning every uAUG located in the 5′ leader sequences of all the 13,051 expressed transcripts. uAUGs with normalized read counts ≥ 23.17 and with raw read counts ≥ 10 in all of the three replicates in the mock condition and/or in response to elf18 were selected and termed ‘translating uAUGs’ (Extended Data Fig. 2a). A total of 5,626 translating uAUGs were identified from the 13,051 expressed transcripts. The remaining 7,968 uAUGs in the 13,051 expressed transcripts are ‘non-translating uAUGs’.

### In vivo SHAPE-MaP in plants and in mammalian cells

The SHAPE reagent, 2-methylnicotinic acid imidazolide (NAI), was synthesized as described previously^63^. For in vivo SHAPE-MaP in plants, *Arabidopsis* seedlings treated with elf18 or water or tobacco leaves transiently expressing the dual-luciferase reporters were collected and immediately immersed in the fresh NAI solution (100 mM NAI) or in dimethyl sulfoxide (DMSO) solution as previously described^64^. To enhance the permeability of NAI, samples immersed in the solution were vacuum-infiltrated and incubated at room temperature for 20 min. To quench the reaction, DTT (dithiothreitol; Roche) was added to the solution for a final concentration of 0.5 M, and incubated for 2 min. The tissue was then washed with water three times, frozen in liquid nitrogen, ground and subjected to total RNA isolation using the Direct-zol RNA Miniprep Plus Kit (Zymo).

For in vivo SHAPE-MaP in the human HEK293FT cell line, cells were collected, washed once with cold 1× PBS after the removal of culture medium and collected in a 1.5-ml tube. Cells were immediately resuspended in 500 μl fresh NAI solution (100 mM NAI) or in 500 μl DMSO solution, and incubated at room temperature with gentle rotation for 5 min. The reaction was stopped by centrifuging the samples at 100,00*g* at 4 °C for 1 min and removing the supernatant. The sample was immediately resuspended in Trizol (Invitrogen) for total RNA isolation using the Direct-zol RNA Miniprep Plus Kit (Zymo).

The purified total RNA from plants or HEK293FT cells was subjected to DNase treatment by adding 2 μl Turbo DNase (2 U μl^−1^) and incubated at 37 °C for 30 min, followed by the addition of another 2 μl Turbo DNase (2 U μl^−1^) and incubation for another 30 min. RNA was then purified by the RNA Clean & Concentrator Kit (Zymo). mRNA was enriched twice through poly(A) selection using Oligo d(T)25 Magnetic Beads (NEB), and subjected to reverse transcription (mRNA in 2.5 μl nuclease-free water, 1 μl 10 mM dNTP (NEB), 1 μl Random Primer 9 (NEB) and 2 μl 5× First-Strand Buffer (Invitrogen), 0.5 μl 0.2 M DTT (Invitrogen), 0.5 μl TGIRT-III (InGex), 0.5 μl SUPERaseIn (Invitrogen) and 2 μl 5 M betaine solution (Sigma-Aldrich)). The cDNA product was cleaned up using the Oligo Clean & Concentrator Kit (Zymo) and the library preparation was performed as described previously^65^, under the randomer library preparation workflow. Agilent 2100 Bioanalyzer was used for the sample quality control. For the global SHAPE-MaP, libraries were pooled and subjected to next-generation sequencing using the Illumina NovaSeq (S4, full flow cell) with paired-end reads of 150 bp in length. For the targeted SHAPE-MaP, gene-specific PCR primers (Supplementary Table 1) were used for the library preparation as described previously^65^, under the amplicon library preparation workflow.

### In vitro SHAPE-MaP in plants

*Arabidopsis* seedlings treated in the mock condition were collected, frozen in liquid nitrogen, ground and subjected to total RNA isolation using the Direct-zol RNA Miniprep Plus Kit (Zymo). The purified RNA was subjected to DNase treatment, clean-up and poly(A) selection as mentioned above. To probe the in vitro RNA secondary structures, 500 ng purified mRNA was mixed with NAI (100 mM) or DMSO in a SHAPE reaction buffer (100 mM HEPES, 6 mM MgCl_2_ and 100 mM NaCl) and incubated at room temperature for 5 min. The reaction was then quenched by purifying RNA using the RNA Clean & Concentrator Kit (Zymo). The treated mRNA was then subjected to reverse transcription, library preparation and next-generation sequencing as described above.

### SHAPE-MaP data processing

For global SHAPE-MaP data processing, raw reads were trimmed with Trim Galore v.0.6.6. The trimmed reads were mapped to the rRNA and tRNA library from the *Arabidopsis* TAIR 10 genome using Bowtie 2 v.2.4.2 (ref. ^55^), and the unmapped reads were aligned to the *Arabidopsis* TAIR 10 transcriptome using Bowtie 2 v.2.4.2 (ref. ^55^). Mapped reads from all four replicates in each group were combined for the following analyses^66,67^: (1) parse the mutations using shapemapper_mutation_parser; (2) count mutation events using shapemapper_mutation_counter; (3) summarize mutation events and calculate SHAPE reactivities using make_reactivity_profiles.py and normalize_profiles.py. Unless specified, only nucleotides with ≥1,000 read coverage and with 0 ≤ SHAPE reactivities ≤ 6 were used for subsequent analyses to ensure accurate structural prediction^30^. To examine the correlation between replicates, SHAPE reactivity for every transcript in each replicate was calculated individually, and the Pearson correlation coefficient for each transcript was determined in R v.4.1.0 using the Hmisc package (https://hbiostat.org/R/Hmisc/). For targeted SHAPE-MaP data processing, raw reads were processed using ShapeMapper 2^66^. To ensure adequate read coverage and completeness, more than 100,000 reads per nucleotide were achieved for more than 90% of the targeted regions. Delta SHAPE reactivity was calculated by taking the log_2_-transformed fold change (elf18/mock) for the SHAPE reactivities of the nucleotide in each position. These values were then smoothed over 10-nt sliding windows^68^. It is worth noting that among the four nucleotides, the increases in mutation rates for adenines in NAI-modified samples were comparably modest (Extended Data Fig. 3d), suggesting that adenine might be less sensitive than the other three residues to NAI modification. However, this does not affect the conclusion of this study, which focuses on identifying the base-pairing status of a region rather than individual nucleotides.

### Training and validation of TISnet

To analyse the structure patterns in downstream regions of initiating AUG, we trained a deep neural network to predict translation initiation sites by adapting the PrismNet model^69^. Downstream regions (101 nt) of mAUGs in transcripts with the top 40% translational efficiency (mAUGs, high likelihood of initiating translation) were used as positive samples and downstream regions of AUGs randomly selected from CDSs or 3′ UTRs (internal AUGs, unlikely to initiate translation) were used as negative samples. Both positive and negative samples must have high SHAPE reactivity coverage (>25%). For the downstream region (101 nt) of each AUG, we predicted RNA secondary structures using RNAfold^70^ with SHAPE reactivity data used as a soft constraint involving a pseudo-free energy calculation under default parameters (the slope ‘m’ is 1.8 and the intercept is –0.6)^71^. Then we trained TISnet to classify initiating and non-initiating AUGs by integrating sequence and secondary structure information.

More specifically, we labelled the positive samples as 1, and negative samples as 0. We then encoded the sequence by one-hot encoding (A, C, G, U, 4-dimension), and encoded RNA secondary structures of each nucleotide to 0 or 1 (0 for nucleotides in double-stranded structures; 1 for nucleotides in single-stranded regions). The labels and encodings of samples were used as the input for the deep neural network. We then randomly split the positive and negative samples into a training set and a validation set by 4:1, and trained the network and validated the prediction performance of the network using the two sets, respectively.

### Identification of structural elements

To find the sequence pattern of hairpin elements, we extracted the hairpin elements with long stems (more than 15 base pairs) from the downstream regions of predicted initiating AUGs. Then we calculated the *k*-mer (*k* = 3) frequency of the loop sequences and the frequency of base pairs in each position (for example, base pairs are counted starting from the loop) of the stem. We further identified conserved structure elements by clustering hairpin elements into classes, on the basis of the sequence similarity between each two hairpin elements. For two sequences, we aligned them by the Needleman–Wunsch algorithm and defined sequence identity as:

$${\rm{Sequence\; identity}}=\,\frac{{\rm{Number\; of\; aligned\; nucleotides}}}{{\rm{Number\; of\; aligned\; and\; unaligned\; nucleotides}}}$$

We divided each hairpin element into 5′ stem sequence (stem-1), loop sequence and 3′ stem sequence (stem-2) (Extended Data Fig. 6c), and calculated the average of sequence identities of these three parts to represent the sequence similarity between two hairpin elements. We calculated the sequence similarity between each two hairpin elements and clustered all hairpin elements in downstream regions of predicted initiating AUGs by the hierarchical clustering algorithm. For each class of hairpin elements, we performed multiple alignment of the stem sequences and the loop sequences and calculated the frequency of nucleotides in each position to construct the position weight matrix (PWM) of the sequence motif. The secondary structures of downstream regions of AUGs were visualized by VARNA^72^.

### 5′ rapid amplification of cDNA ends

For the 5′ rapid amplification of cDNA ends (RACE) experiment on the RNA products from all the constructs expressed in plants, a FLUC-specific reverse transcription primer (Supplementary Table 1) and the Template Switching RT Enzyme Mix (NEB) were used during cDNA synthesis; this was followed by template switching using the Template Switching Oligo. PCR amplification of the 5′ region of transcripts was performed using Q5 Hot Start High-Fidelity Master Mix (2×) (NEB).

### In vitro transcription

For in vitro transcription, the PCR product containing a T7 RNA polymerase promoter (GCTAATACGACTCACTATAGGG) was used to generate mRNA by using the mMESSAGE mMACHINE T7 ULTRA Transcription Kit (Ambion, AM1344) according to the manufacturer’s instructions. The mRNA product was purified using the MEGAclear Transcription Clean-Up Kit (Ambion, AM1908). To validate the quality of the mRNA product, samples were run on 1% denaturing agarose gel and stained with SYBR Gold (Invitrogen).

### Dual-luciferase assay

The dual-luciferase assay for plant samples was performed as described^20^. In brief, an overnight culture of the *Agrobacterium* strain GV3101 transformed with the dual-luciferase construct was collected, resuspended in the infiltration buffer (10 mM MgCl_2_, 10 mM MES and 200 μM acetosyringone), adjusted to an optical density at 600 nm (OD_600 nm_) of 0.2 and incubated at room temperature for an additional 2 h before infiltrating into *N. benthamiana* for transient expression. After 24 h of incubation, leaf discs were collected, ground in liquid nitrogen and lysed with 1× passive lysis buffer (Promega). The lysate was centrifuged at 12,000*g* for 3 min, and 10 μl supernatant was used for measuring FLUC and RLUC activities as previously described^20^. For the experiment with dex-induced expression, the *Agrobacterium* strain with the dual-luciferase construct and the strain with the dex-inducible RNA helicase construct were co-infiltrated into *N. benthamiana* leaves and incubated for 20 h. Then, the leaves were sprayed with 25 μM dex solution in water and incubated for another 4 h before sample collection.

The dual-luciferase assay in the human cell line was performed according to the manufacturer’s instructions (Promega). In brief, HEK293FT cells were seeded into 96-well plates and grown overnight to approximately 70% confluence at the time of transfection. Then, 100 ng of FLUC mRNAs and 100 ng of RLUC mRNAs were co-transfected into HEK293FT cells using 0.3 µl Lipofectamine MessengerMAX Transfection Reagent (Invitrogen) for each well. After a 5-h incubation, cells were collected and washed once with cold 1× PBS after the removal of the culture medium. Fifty microlitres of 1× passive lysis buffer (Promega) was used to extract the proteins according to standard procedures, and 10 µl lysate was used for measuring FLUC and RLUC activities as previously described^20^.

### Western blotting assay

To detect the dex-induced YFP-tagged proteins, the blot was probed with anti-GFP (Clontech, 632381, 1:5,000) primary antibodies. To detect HA-tagged proteins, the blot was probed with anti-HA HRP-conjugated antibody (Cell Signaling Tech, 2999, 1:3,000). To detect endogenous proteins, the blot was probed with anti-ARF2 primary antibody (PhytoAB, PHY2435A, 1:2,000), anti-CH1 primary antibody (PhytoAB, PHY1909S, 1:2,000), anti-RBOHD primary antibody (Agrisera, AS15 2962, 1:2,000), anti-ICS1 primary antibody (Agrisera, AS16 4107, 1:2,000) or anti-β-tubulin primary antibody (Santa Cruz Biotech, sc-166729, 1:2,000). For secondary antibodies, anti-rabbit-HRP antibody (Cell Signaling Tech, 7074, 1:3,000) or anti-mouse-HRP antibody (Abcam, Ab97040, 1:10,000) were used.

### Elf18-induced resistance to *Psm* ES4326

The elf18-induced resistance experiment was performed as previously described^20^. In brief, *Arabidopsis* plants were grown in soil for three to four weeks and infiltrated with 1 μM elf18 or mock treatment (water) one day before infection with *Psm* ES4326 (in 10 mM MgCl_2_ solution at OD_600 nm_ = 0.001) in the same leaf. Bacterial growth was measured two days after infection.

### Statistics and reproducibility

Unless specified, statistical tests were performed using GraphPad Prism v.8.0 or in R v.4.1.0. The statistical methods and number of experimental replicates are indicated in the figure legends. Unless specified in the figures or legends, no adjustments were made for multiple comparisons. In the graphs (except for Fig. 3b,c), asterisks and lower-case letters indicate statistical significance reflecting the *P* values (*P < 0.05, **P < 0.01, ***P < 0.001, ****P < 0.0001; NS, not significant). The number of data points for the analyses shown in Figs. 2c,d and 3g and Extended Data Fig. 4d are as follows: upstream, *n* = 50; downstream, *n* = 50. For Fig. 2e, m/iAUG, predicted non-initiating AUG, *n* = 7,083; predicted initiating AUG, *n* = 2,917; uAUG, predicted non-initiating AUG, *n* = 895; predicted initiating AUG, *n* = 933. For Fig. 2i, only transcripts with high expression levels (RPKM > 19) were used for the analysis. Predicted non-initiating AUG, *n* = 450; predicted initiating AUG, *n* = 464. For Fig. 4e, WT, *n* = 50; *rh37* *rh52*, *n* = 50. For Extended Data Fig. 4c,e, in vivo, *n* = 50; in vitro, *n* = 50. Unless specified, experiments were repeated at least three times with similar results. Original gel images can be found in Supplementary Fig. 1.

### Reporting summary

Further information on research design is available in the Nature Portfolio Reporting Summary linked to this article.

## Online content

Any methods, additional references, Nature Portfolio reporting summaries, source data, extended data, supplementary information, acknowledgements, peer review information; details of author contributions and competing interests; and statements of data and code availability are available at 10.1038/s41586-023-06500-y.

## Supplementary information

Supplementary Figure 1 Full scans of the gel images.

Reporting Summary

Supplementary Table 1 A list of primers, oligos and synthesized DNA used in the study.

## Data Availability

The Ribo-seq, RNA-seq and SHAPE-MaP sequencing data are available through the National Center for Biotechnology Information (NCBI) under accession number PRJNA852547.

## Code availability

Code for the TISnet model and further analysis are available at https://github.com/huangwenze/TISnet.
