# Supplementary material for: Pervasive downstream RNA hairpins dynamically dictate start-codon selection
Source: Nature. 2023 Sep 6;621(7978):423–30. doi: 10.1038/s41586-023-06500-y (PMC10499604; doi:10.1038/s41586-023-06500-y)
Supplement: Supplementary file 2 — Reporting Summary [file 41586_2023_6500_MOESM2_ESM.pdf]

nature portfolio

Corresponding author(s): Xinnian Dong

Last updated by author(s): Jul 11, 2023

Reporting Summary

Nature Portfolio wishes to improve the reproducibility of the work that we publish. This form provides structure for consistency and transparency in reporting. For further information on Nature Portfolio policies, see our Editorial Policies and the Editorial Policy Checklist.

Statistics

For all statistical analyses, confirm that the following items are present in the figure legend, table legend, main text, or Methods section.

n/a	Confirmed	
☐	☒	The exact sample size (*n*) for each experimental group/condition, given as a discrete number and unit of measurement
☐	☒	A statement on whether measurements were taken from distinct samples or whether the same sample was measured repeatedly
☐	☒	The statistical test(s) used AND whether they are one- or two-sided<br>*Only common tests should be described solely by name; describe more complex techniques in the Methods section.*
☒	☐	A description of all covariates tested
☒	☐	A description of any assumptions or corrections, such as tests of normality and adjustment for multiple comparisons
☐	☒	A full description of the statistical parameters including central tendency (e.g. means) or other basic estimates (e.g. regression coefficient) AND variation (e.g. standard deviation) or associated estimates of uncertainty (e.g. confidence intervals)
☐	☒	For null hypothesis testing, the test statistic (e.g. *F*, *t*, *r*) with confidence intervals, effect sizes, degrees of freedom and *P* value noted<br>*Give P values as exact values whenever suitable.*
☒	☐	For Bayesian analysis, information on the choice of priors and Markov chain Monte Carlo settings
☒	☐	For hierarchical and complex designs, identification of the appropriate level for tests and full reporting of outcomes
☐	☒	Estimates of effect sizes (e.g. Cohen's *d*, Pearson's *r*), indicating how they were calculated

Our web collection on statistics for biologists contains articles on many of the points above.

Software and code

Policy information about availability of computer code

Data collection	Victor3 plate reader (PerkinElmer) was used to measure FLUC and RLUC activity
Data analysis	Trim Galore v0.6.6; Bowtie 2 v2.4.2; STAR v2.7.8a; FastQC v0.11.9; MultiQC v1.9; Plastid v0.6.1; deltaTE; Gene Ontology resource; ShapeMapper 2; ViennaRNA v2.0; RNAfold; VARNA; R v4.1.0; ggplot2 v3.3.5; dplyr v1.0.8; tidyverse v1.3.1; stringr v1.4.0; https://github.com/huangwenze/TISnet.

For manuscripts utilizing custom algorithms or software that are central to the research but not yet described in published literature, software must be made available to editors and reviewers. We strongly encourage code deposition in a community repository (e.g. GitHub). See the Nature Portfolio guidelines for submitting code & software for further information.

Data

Policy information about availability of data

All manuscripts must include a data availability statement. This statement should provide the following information, where applicable:

- Accession codes, unique identifiers, or web links for publicly available datasets
- A description of any restrictions on data availability
- For clinical datasets or third party data, please ensure that the statement adheres to our policy

The Ribo-seq, RNA-seq, and SHAPE-MaP data have been deposited to the NCBI under the BioProject ID PRJNA852547.

March 2021

Human research participants

Policy information about studies involving human research participants and Sex and Gender in Research.

Reporting on sex and gender	NA
Population characteristics	NA
Recruitment	NA
Ethics oversight	NA

Note that full information on the approval of the study protocol must also be provided in the manuscript.

Field-specific reporting

Please select the one below that is the best fit for your research. If you are not sure, read the appropriate sections before making your selection.

☒ Life sciences ☐ Behavioural & social sciences ☐ Ecological, evolutionary & environmental sciences

For a reference copy of the document with all sections, see nature.com/documents/nr-reporting-summary-flat.pdf

Life sciences study design

All studies must disclose on these points even when the disclosure is negative.

Sample size	No sample size calculation was performed. Sample sizes were chosen according to the established protocols or preliminary results. At least five plants were sampled for each biological replicate. Biological repeats were pooled for statistical tests.
Data exclusions	No data were excluded from the analysis.
Replication	All the experiments have at least three biological replicates, and/or were repeated for at least twice. All the experimental findings were reproducible.
Randomization	Sample allocation was random.
Blinding	No blinding was used during experimentation because plants with different genetic backgrounds needed to be identified for the different treatments.

Reporting for specific materials, systems and methods

We require information from authors about some types of materials, experimental systems and methods used in many studies. Here, indicate whether each material, system or method listed is relevant to your study. If you are not sure if a list item applies to your research, read the appropriate section before selecting a response.

Materials & experimental systems

n/a	Involved in the study	
☐	☒	Antibodies
☐	☒	Eukaryotic cell lines
☒	☐	Palaeontology and archaeology
☒	☐	Animals and other organisms
☒	☐	Clinical data
☒	☐	Dual use research of concern

Methods

n/a	Involved in the study	
☒	☐	ChIP-seq
☒	☐	Flow cytometry
☒	☐	MRI-based neuroimaging

Antibodies

Antibodies used	(1) anti-GFP: Living Colors® A.v. Monoclonal Antibody (JL-8), Takara Bio Clontech (#632381)<br>(2) anti-mouse-HRP: Goat Anti-Mouse IgG H&L (HRP), Abcam (#ab97040)<br>(3) anti-HA: HA-Tag (6E2) Mouse mAb (HRP Conjugate), Cell Signaling Technology, Inc (#2999)<br>(4) anti-ARF2: Anti-Auxin Response Factor 2 Antibody, PHYTOAB (#PHY2435A)<br>(5) anti-CAO/CH1: Anti-Chlorophyllide A Oxygenase, Chloroplastic Antibody, PHYTOAB (#PHY1909S)

(6) anti-RBOHD: Anti-Respiratory burst oxidase homolog protein D Antibody, Agrisera (#AS15 2962)
(7) anti-ICS1: Anti-Protein SAR DEFICIENT 1 Antibody, Agrisera (#AS16 4107)
(8) anti-beta-tubulin: beta Tubulin Antibody, Santa Cruz Biotech (#sc-166729)
(9) anti-rabbit-HRP: Goat Anti-Rabbit IgG H&L (HRP), Cell Signaling Tech(#7074)

Validation

(1) anti-GFP: https://www.takarabio.com/documents/Certificate%20of%20Analysis/632380/632380-632381-070313.pdf
(2) anti-mouse-HRP: https://www.abcam.com/goat-mouse-igg-hl-hrp-preadsorbed-ab97040.html
(3) anti-HA: https://media.cellsignal.com/coa/2999/4/2999-lot-4-coa.pdf
(4) anti-ARF2: https://www.phytoab.com/mwdownloads/download/link/id/3653
(5) anti-CAO/CH1: https://www.phytoab.com/mwdownloads/download/link/id/4636
(6) anti-RBOHD: https://www.agrisera.com/cgi-bin/ibutik/SkapaFaktura.pl?SkrivPDF=J&Friendly_Grupp=&skrivpdf=j&artgrp=112&funk=visa_artikel&Friendly=rbohd-respiratory-burst-oxidase-homolog-protein-d-&artnr=AS15%202962&Sprak=EN
(7) anti-ICS1: https://www.agrisera.com/cgi-bin/ibutik/SkapaFaktura.pl?SkrivPDF=J&funk=visa_artikel&artnr=AS16%204107&Sprak=EN&skrivpdf=j&Friendly_Grupp=&artgrp=116&Friendly=ics1-isochorismate-synthase-1-chloroplastic
(8) anti-beta-tubulin: https://datasheets.scbt.com/sc-166729.pdf
(9) anti-rabbit-HRP: https://media.cellsignal.com/coa/7074/32/7074-lot-32-coa.pdf

Eukaryotic cell lines

Policy information about cell lines and Sex and Gender in Research

Cell line source(s)	HEK293FT cell line was purchased from Duke Cell Culture Facility, Invitrogen Cat# R700-07
Authentication	Cell line identity was confirmed by STR Authentication.
Mycoplasma contamination	All cells were tested negative for mycoplasma contamination.
Commonly misidentified lines (See ICLAC register)	No commonly misidentified cell lines were used.
